# Women’s Adherence to Healthy Dietary Patterns and Outcomes of Infertility Treatment

**DOI:** 10.1001/jamanetworkopen.2023.29982

**Published:** 2023-08-18

**Authors:** Albert Salas-Huetos, Makiko Mitsunami, Siwen Wang, Lidia Mínguez-Alarcón, Jordi Ribas-Maynou, Marc Yeste, Irene Souter, Jorge E. Chavarro

**Affiliations:** 1 Department of Nutrition, Harvard T. H. Chan School of Public Health, Boston, Massachusetts; 2 Unit of Preventive Medicine and Public Health, Alimentació, Nutrició, Desenvolupament i Salut Mental, Faculty of Medicine and Health Sciences, Institut d'Investigació Sanitària Pere Virgili, Universitat Rovira i Virgili, Reus, Spain; 3 Consorcio Centro de Investigación Biomédica en Red, M.P. Fisiopatología de la Obesidad y Nutrición, Instituto de Salud Carlos III, Madrid, Spain; 4 Channing Division of Network Medicine, Department of Medicine, Brigham and Women’s Hospital and Harvard Medical School, Boston, Massachusetts; 5 Department of Environmental Health, Harvard T. H. Chan School of Public Health, Boston, Massachusetts; 6 Biotechnology of Animal and Human Reproduction (TechnoSperm), Institute of Food and Agricultural Technology, University of Girona, Girona, Spain.; 7 Unit of Cell Biology, Faculty of Sciences, Department of Biology, University of Girona, Girona, Spain; 8 Massachusetts General Hospital Fertility Center and Harvard Medical School, Boston; 9 Department of Epidemiology, Harvard T. H. Chan School of Public Health, Boston, Massachusetts

## Abstract

**Importance:**

Increasing evidence suggests that specific foods and nutrients may improve infertility treatment outcomes in women. However, less is known about the role of dietary patterns.

**Objective:**

To investigate whether women’s adherence to a priori–defined dietary patterns promoted for the prevention of chronic conditions is associated with outcomes of infertility treatment.

**Design, Setting, and Participants:**

This prospective cohort study was conducted at a fertility center at an academic medical center in Boston, Massachusetts. Women undergoing infertility treatment cycles, including intrauterine insemination cycles and in vitro fertilization with or without intracytoplasmic sperm injection were included. Data were collected from January 2007 to October 2019, and data were analyzed from February to December 2022.

**Exposures:**

Women’s pretreatment diet was assessed with a validated food frequency questionnaire from which 8 a priori–defined scores were calculated (higher score indicates greater adherence): (1) Trichopoulou Mediterranean diet, (2) alternate Mediterranean diet, (3) Panagiotakos Mediterranean diet, (4) Healthy Eating Index, (5) Alternate Healthy Eating Index, (6) American Heart Association (AHA) index, (7) Dietary Approaches to Stop Hypertension index, and (8) plant-based diet.

**Main Outcomes and Measures:**

The adjusted probability of clinically relevant outcomes (live birth as a primary outcome and clinical pregnancy and pregnancy loss as secondary outcomes) was evaluated across quartiles of adherence to each dietary pattern using multivariable generalized linear mixed models to account for repeated cycles.

**Results:**

This analysis included 612 women with a median (IQR) age of 35.0 (32.0-38.0) years. There was no association between women’s adherence to the 8 a priori dietary patterns and probability of clinical pregnancy or live birth following in vitro fertilization or intrauterine insemination. However, an inverse association was found between adherence to AHA dietary pattern and risks of total and clinical pregnancy loss. Among women who became pregnant during the course of infertility treatment, the adjusted probabilities of pregnancy loss in the lowest and highest quartile of the AHA dietary pattern were 0.41 (95% CI, 0.33-0.50) and 0.28 (95% CI, 0.21–0.36), respectively (*P* for trend = .02). The corresponding adjusted probabilities of clinical pregnancy loss were 0.30 (95% CI, 0.22-0.39) and 0.15 (95% CI, 0.10-0.23) (*P* for trend = .007). A similar pattern was observed for all other dietary patterns, with the exception of the plant-based diet pattern.

**Conclusions and Relevance:**

Findings of this cohort study suggest that preconception adherence to the AHA diet may be associated with a lower likelihood of pregnancy loss during the course of infertility treatment.

## Key Points

**Question:**

Is adherence to dietary patterns promoted for the prevention of cardiovascular disease and other chronic conditions associated with the outcome of infertility treatment?

**Findings:**

In this cohort study of 612 women who underwent 804 intrauterine insemination cycles and 768 in vitro fertilization, higher adherence to the American Heart Association index was inversely associated with pregnancy loss. Similar associations were observed with the Dietary Approaches to Stop Hypertension diet, the Healthy Eating Index, and Alternate Healthy Eating Index and the Mediterranean diet.

**Meaning:**

Adherence to preconception healthy dietary patterns before infertility treatment may be associated with a lower likelihood of pregnancy loss.

## Teaser

This cohort study evaluates whether women’s adherence to dietary patterns promoted for the prevention of chronic conditions is associated with outcomes of infertility treatment.

## Introduction

Infertility is a widespread problem for couples worldwide, with an estimated prevalence of 12% to 15%.^1,2^ The increasing trend in infertility observed in recent decades is potentially influenced by lifestyle and environmental factors,^3,4,5^ including air pollution and obesity, among others.^6^ Furthermore, mounting evidence suggests that couples’ diet may influence fertility.^7,8^ Although evidence is still scant, there is also some evidence suggesting that diet may have an impact on outcomes of infertility treatment. Vujkovic and colleagues^9^ reported that a data-derived dietary pattern characterized by high intakes of vegetable oils, vegetables, fish, and legumes and low intakes of snacks was associated with a higher probability of pregnancy during infertility treatment with in vitro fertilization (IVF). We have previously reported that adherence to a diet rich in folic acid, vitamin B12, vitamin D, low-pesticide fruits and vegetables, whole grains, seafood, dairy, and soy foods is associated with a higher likelihood of live birth following infertility treatment with assisted reproductive technologies (ART).^10^ Similarly, Karayiannis and collaborators^11^ reported that greater adherence to a Mediterranean dietary pattern was related to a higher probability of live birth during IVF. A separate group reported that a short-term intervention aimed at increasing adherence to a Mediterranean dietary pattern has a significant effect on embryo cleavage rates.^12^

Nevertheless, scoring systems to define adherence to specific dietary patterns are not consistent across studies, and more often than not, studies do not address how adherence to several different patterns impacts outcomes of infertility treatments, resulting in apparently contradictory findings. The present study aimed to investigate whether women’s adherence to 8 commonly used, a priori–defined dietary patterns originally designed to assess the role of diet on cardiovascular disease and other chronic conditions are associated with outcomes of infertility treatment (pregnancy loss, clinical pregnancy, and live birth). Based on findings from previous studies, we hypothesized that adherence to healthy dietary patterns would be associated with higher live birth rates and that this association would be partly a reflection of a lower frequency of pregnancy loss.

## Methods

### Study Population

Women aged 18 to 45 years presenting for infertility evaluation and treatment to the Massachusetts General Hospital (MGH) Fertility Center were invited to enroll in a prospective preconception cohort, the Environment and Reproductive Health (EARTH) study. Established in 2004, the EARTH study aimed to identify environmental and nutritional determinants of fertility.^13^ Included women completed several study questionnaires at baseline, including demographic characteristics (eg, self-reported race and ethnicity and education level via a questionnaire), medical and reproductive history, lifestyle (eg, physical activity^14^), and diet (introduced in 2007) and underwent an anthropometric evaluation. Participants were asked to report their race as American Indian or Alaska Native, Asian, Black, Native Hawaiian or Other Pacific Islander, White, or other race, in which case they were asked to specify. They were also separately asked whether they identified as Hispanic. Between 2007 and 2019, 728 women enrolled in the study. Of these, 17 (2.4%) discontinued care at MGH, and it was therefore not possible to assess any outcomes. Of the remaining 711 women, 99 did not complete a baseline dietary assessment, leaving 612 women in the analytical sample. These women completed a total of 1572 treatment cycles, including 302 women who completed 804 intrauterine insemination (IUI) cycles and 450 women who completed 768 IVF cycles (eFigure 1 in Supplement 1). The 99 women excluded from the analysis had similar age and body mass index (BMI; calculated as weight in kilograms divided by height in meters squared) to women included in the analysis but were less likely to be non-Hispanic White (71 of 99 [74%] vs 505 of 612 [83%]), less likely to have ever smoked (21 [22%] vs 151 [25%]), and more likely to have completed college (36 [95%] vs 538 [92%]). All participants provided written informed consent. Clinical information, including treatment outcomes, was abstracted from the patient’s electronic medical records. The study protocol was approved by the institutional review boards of the MGH and the Harvard T. H. Chan School of Public Health. This study followed the Strengthening the Reporting of Observational Studies in Epidemiology (STROBE) reporting guideline.

### Covariate and Exposure Assessment

Several general and anthropometric variables were determined by trained study staff at enrollment. Height and weight were assessed to calculate BMI. Moreover, detailed take-home questionnaires regarding lifestyle factors (eg, smoking), medical history, physical activity,^14^ and reproductive health were completed by included participants. The main exposure of this study was the women’s preconception diet assessed using an extensively validated semiquantitative food frequency questionnaire with more than 131 foods and beverages.^15,16^ Women were asked to report how often, on average, during the previous year they consumed each item. We assessed the adherence to 8 a priori–defined dietary pattern scores: (1) Trichopoulou Mediterranean diet (TMD),^17^ (2) alternate Mediterranean diet (AMD),^18^ (3) Panagiotakos Mediterranean diet (PMD),^19^ (4) Healthy Eating Index (HEI),^20^ (5) Alternate Healthy Eating Index (AHEI),^21^ (6) American Heart Association (AHA) 2020 dietary goals index,^22^ (7) Dietary Approaches to Stop Hypertension (DASH) index,^23^ and (8) plant-based diet (PBD).^24^ Briefly, the PBD encourages the consumption of plant-based foods at the expense of all animal products. While differing in specific cutoffs and food groupings, all other patterns encourage the consumption of fruits, vegetables, nuts, legumes, whole grains, fish, and olive oil or monounsaturated fats while discouraging the intake of red meats. Some patterns also incorporate intake of alcohol (moderate intake generally encouraged), sodium (intake discouraged), saturated fat (intake discouraged), and sugary beverages (intake discouraged). Higher scores indicate higher adherence to the respective diet patterns. eTable 1 in Supplement 1 summarizes the different components and scoring criteria for each dietary pattern.

### Outcome Assessment

The primary outcome of this study was live birth per initiated treatment cycle. Secondary outcomes included clinical pregnancy, total pregnancy loss, and clinical pregnancy loss. Pregnancy was defined by a serum β-human chorionic gonadotropin (β-hCG) level greater than 6 mIU/mL, clinical pregnancy by the presence of an intrauterine gestational sac confirmed by ultrasonography at 6 weeks’ gestation, and live birth by a neonate birth at or after 24 weeks’ gestation. Total pregnancy loss was defined as β-hCG greater than 6 mIU/mL that did not result in live births (thus including biochemical and clinical pregnancy losses), and clinical pregnancy loss was defined as a clinical pregnancy that did not result in a live birth. During stimulation, women were also monitored for serum estradiol and endometrial thickness, both considered early ART outcomes.

### Statistical Analysis

Participants were categorized into quartiles of adherence to the 8 dietary scores using the lowest quartile as the reference group. Baseline demographic, dietary, and reproductive characteristics of study participants were reported as medians with IQRs for continuous variables or counts with percentages for categorical variables. Baseline differences were compared across quartiles of dietary patterns using the Kruskal-Wallis test for continuous variables and χ^2^ test for categorical variables. Spearman correlations were used to describe similarities between the dietary pattern scores evaluated.

Multivariable generalized linear mixed models were used to assess the association between exposure (dietary patterns) and outcomes (clinical pregnancy, live birth, total pregnancy loss, and clinical pregnancy loss) with binomial or binary distribution and random intercepts to account for repeated cycles. Tests for linear trends across quartiles of adherence were conducted using the median of each dietary pattern score as a continuous variable in the regression models. Results are presented as predicted marginal proportions and 95% CIs. Since success rates differ substantially between IUI and IVF cycles, analyses evaluating associations with clinical pregnancy and live birth were conducted separately for IUI and IVF cycles.^25,26^ However, since the probability of pregnancy loss is comparable in IUI and IVF cycles,^27^ analyses focused on pregnancy loss included analyses of IUI and IVF cycles separately as well as analyses combining both IUI and IVF cycles.

Confounding factors were evaluated using previous knowledge and analyzing the association of each of the descriptive variables from our cohort with dietary patterns quartiles using Kruskal-Wallis tests for continuous variables or χ^2^ tests for categorical variables. Multivariable adjusted models included female age, BMI, physical activity, and energy intake. We conducted different sensitivity analyses to evaluate the robustness of the findings. First, we compared the main associations in IVF cycles with those restricting the analysis to couples without cycle cancelations. Those models were additionally adjusted for number of embryos transferred (1, 2, or 3 or more) and day of embryo transfer (day 2, day 3, or day 5). Second, we compared the main associations combining both IUI and IVF cycles with those after exclusion of egg donor cycles.

All statistical analyses were performed using SAS version 9.4 (SAS Institute). *P* values less than .05 were considered statistically significant, and all *P* values were 2-tailed.

## Results

This analysis included 612 women who underwent 1572 infertility treatment cycles, including 804 IUI cycles and 768 IVF cycles. Participants had a median (IQR) age of 35.0 (32.0-38.0) years and median (IQR) BMI of 23.4 (21.3-26.4). A total of 59 (9.7%) were Asian, 26 (4.3%) were Black, 505 (82.8%) were White, and 20 (3.3%) were another race or ethnicity (including American Indian or Alaska Native, Native Hawaiian or Other Pacific Islander, and other race). Most women had never smoked (461 [75.3%]) and had a college degree (538 [92.1%]). Female factor infertility was the most common initial primary infertility diagnosis (235 of 607 [38.7%]). Among women who underwent IVF cycles, luteal-phase gonadotropin-releasing hormone agonist protocol was the most used initial stimulation protocol (283 of 450 [62.9%]), day 5 was the most common embryo transfer day (168 of 345 [48.7%]), and 2 embryos were transferred in most of the ART cycles (183 of 345 [52.9%]) (Table 1). Baseline reproductive characteristics of study participants, overall and in the lowest and highest quartiles of women’s adherence to the different dietary patterns who underwent infertility treatment with IUI or IVF, are shown in eTables 2 to 4 in Supplement 1. All dietary patterns were modestly to highly correlated with one another (ρ range, 0.35-0.88), with the highest correlation observed between the TMD and AMD scores and the lowest correlation observed between the AHEI and PBD score (eFigure 2 in Supplement 1).

**Table 1.** Baseline Demographic and Reproductive Characteristics of Study Participants Who Underwent Infertility Treatment (Intrauterine Insemination [IUI] or In Vitro Fertilization [IVF])

Characteristic	Overall, No. (%)		
	IVF	IUI	IVF and IUI
Total, No.	450	302	612
Female demographic characteristics			
Age, median (IQR), y	35.0 (32.0-39.0)	34.0 (32.0-37.5)	35.0 (32.0-38.0)
BMI, median (IQR)^a^	23.2 (21.2-26.0)	23.7 (21.3-26.6)	23.4 (21.3-26.4)
Race and ethnicity^b^			
Asian	46 (10.3)	28 (9.3)	59 (9.7)
Black	17 (3.8)	14 (4.7)	26 (4.3)
White	372 (83.0)	244 (81.3)	505 (82.8)
Other race or ethnicity	13 (2.9)	14 (4.7)	20 (3.3)
Ever smoker	107 (23.8)	81 (26.9)	151 (24.7)
College education or higher	398 (92.3)	269 (93.4)	538 (92.1)
Moderate to vigorous physical activity, median (IQR), min/wk^c^	128.5 (29.5-300.0)	150.0 (41.5-314.5)	135.5 (29.5-308.0)
Female energy intake, median (IQR), kcal/d	1687 (1354-2086)	1646 (1356-2037)	1688 (1364-2076)
Couple cycle characteristics			
Infertility diagnosis			
Female factor	153 (34.0)	48 (16.2)	235 (38.7)
Male factor	153 (34.0)	125 (42.1)	178 (29.1)
Unexplained	124 (27.6)	124 (41.8)	194 (32.0)
Treatment protocol			
Antagonist	68 (15.1)	NA	48 (7.8)
Flare^d^	50 (11.1)	NA	41 (6.7)
Luteal phase agonist^e^	283 (62.9)	NA	484 (79.1)
Egg donor or cryo cycle	49 (10.9)	NA	39 (6.4)
Male demographic characteristics			
Age, median (IQR), y	36.5 (32.9-40.0)	35.2 (32.4-38.8)	36.3 (32.7-39.8)
BMI, median (IQR)^a^	26.9 (24.3-29.3)	27.8 (24.7-30.8)	27.1 (24.4-29.8)
Race and ethnicity^b^			
Asian	16 (5.9)	7 (4.7)	20 (5.8)
Black	6 (2.2)	5 (3.4)	10 (2.9)
White	240 (88.9)	134 (90.5)	307 (88.5)
Other race or ethnicity	8 (3.0)	2 (1.4)	10 (2.9)
Ever smoker	86 (31.7)	49 (33.1)	113 (32.5)
College education or higher	188 (85.1)	110 (88.0)	244 (85.6)
Moderate to vigorous physical activity, median (IQR), min/wk^c^	150.0 (0-372.0)	150.0 (12.0-371.5)	149.5 (0-369.8)

Abbreviations: BMI, body mass index; NA, not applicable.

^a^ Calculated as weight in kilograms divided by height in meters squared.

^b^ Race and ethnicity data were self-reported via a questionnaire. The other race and ethnicity category includes American Indian or Alaska Native, Native Hawaiian or Other Pacific Islander, and other race.

^c^ Includes weight and aerobic exercise and sports.

^d^ Follicular-phase gonadotropin-releasing hormone agonist/flare protocol.

^e^ Luteal-phase gonadotropin-releasing hormone agonist protocol.

There was no association between women’s adherence to any of the 8 a priori–defined dietary patterns and probability of clinical pregnancy or live birth following infertility treatment with IVF or IUI when evaluated separately (Table 2) or when combining data from IVF and IUI cycles (eTable 5 in Supplement 1). Results for IVF cycles were similar when analyses were restricted to couples without cycle cancelations, which included additional adjustment for number of embryos transferred and embryo transfer day (eTable 6 in Supplement 1). A sensitivity analysis excluding egg donor cycles was consistent with the primary analysis (eTable 7 in Supplement 1). When ART early outcomes (estradiol trigger levels and endometrial thickness) were explored, we only found a marginal association between higher adherence to the PMD score and lower estradiol trigger levels (eTable 8 in Supplement 1).

**Table 2.** Association Between Women’s Adherence to A Priori–Defined Dietary Patterns According to Quartiles of Distribution and Probability of Clinical Pregnancy and Live Birth Following In Vitro Fertilization (IVF) or Intrauterine Insemination (IUI)^a^

Quartile	IVF,			IUI		
	Women, No.; cycles, No.	Adjusted proportions (95% CI)^b^		Women, No.; cycles, No.	Adjusted proportions (95% CI)^c^	
		Clinical pregnancy	Live birth		Clinical pregnancy	Live birth
Total, No.	450; 768	NA	NA	302; 804	NA	NA
TMD						
1	141; 245	0.49 (0.43-0.56)	0.40 (0.33-0.47)	91; 251	0.17 (0.12-0.22)	0.11 (0.07-0.15)
2	84; 146	0.48 (0.40-0.57)	0.39 (0.31-0.48)	55; 139	0.11 (0.07-0.18)	0.08 (0.04-0.14)
3	84; 145	0.52 (0.44-0.61)	0.42 (0.34-0.51)	108; 275	0.13 (0.09-0.17)	0.09 (0.06-0.14)
4	141; 232	0.51 (0.44-0.57)	0.43 (0.36-0.50)	48; 139	0.14 (0.09-0.21)	0.12 (0.07-0.19)
*P* for trend	NA	.71	.58	NA	.32	.79
AMD						
1	86; 152	0.49 (0.41-0.57)	0.41 (0.33-0.50)	56; 156	0.16 (0.11-0.23)	0.09 (0.05-0.15)
2	142; 236	0.52 (0.45-0.58)	0.40 (0.34-0.47)	104; 263	0.15 (0.11-0.20)	0.11 (0.07-0.15)
3	94; 171	0.47 (0.40-0.55)	0.41 (0.33-0.49)	51; 128	0.13 (0.08-0.20)	0.10 (0.06-0.16)
4	128; 209	0.52 (0.45-0.59)	0.43 (0.36-0.50)	91; 257	0.12 (0.09-0.17)	0.10 (0.06-0.14)
*P* for trend	NA	.74	.71	NA	.32	.99
PMD						
1	104; 194	0.47 (0.39-0.54)	0.36 (0.29-0.43)	78; 227	0.15 (0.11-0.20)	0.10 (0.06-0.14)
2	106; 167	0.52 (0.44-0.60)	0.44 (0.36-0.53)	78; 172	0.17 (0.12-0.24)	0.12 (0.08-0.18)
3	130; 222	0.52 (0.46-0.59)	0.43 (0.36-0.50)	70; 181	0.10 (0.06-0.15)	0.08 (0.05-0.13)
4	110; 185	0.50 (0.43-0.58)	0.42 (0.34-0.49)	76; 224	0.14 (0.10-0.19)	0.10 (0.07-0.15)
*P* for trend	NA	.46	.33	NA	.47	.84
HEI						
1	107; 193	0.51 (0.44-0.58)	0.41 (0.34-0.48)	68; 200	0.15 (0.11-0.21)	0.09 (0.06-0.14)
2	113; 191	0.49 (0.42-0.57)	0.41 (0.33-0.49)	71; 202	0.11 (0.08-0.17)	0.10 (0.07-0.15)
3	110; 190	0.50 (0.43-0.57)	0.41 (0.34-0.49)	82; 202	0.13 (0.09-0.19)	0.10 (0.07-0.15)
4	120; 194	0.51 (0.43-0.58)	0.42 (0.35-0.50)	81; 200	0.16 (0.11-0.21)	0.10 (0.07-0.16)
*P* for trend	NA	.99	.80	NA	.93	.66
AHEI						
1	112; 195	0.54 (0.47-0.61)	0.43 (0.36-0.51)	67; 194	0.16 (0.12-0.22)	0.11 (0.07-0.16)
2	117; 193	0.52 (0.45-0.60)	0.43 (0.36-0.50)	77; 213	0.12 (0.08-0.17)	0.09 (0.05-0.13)
3	105; 195	0.46 (0.39-0.53)	0.38 (0.31-0.45)	78; 191	0.12 (0.08-0.18)	0.08 (0.05-0.13)
4	116; 185	0.48 (0.41-0.56)	0.41 (0.33-0.48)	80; 206	0.15 (0.10-0.21)	0.12 (0.08-0.18)
*P* for trend	NA	.16	.42	NA	.69	.68
AHA index						
1	108; 201	0.46 (0.39-0.53)	0.34 (0.28-0.41)	68; 191	0.16 (0.11-0.22)	0.09 (0.06-0.14)
2	105; 176	0.55 (0.47-0.62)	0.45 (0.37-0.53)^d^	86; 218	0.13 (0.09-0.18)	0.11 (0.07-0.16)
3	129; 108	0.53 (0.46-0.60)	0.44 (0.37-0.52)	76; 199	0.10 (0.07-0.16)	0.08 (0.05-0.13)
4	108; 183	0.48 (0.40-0.55)	0.41 (0.34-0.49)	72; 196	0.16 (0.11-0.22)	0.12 (0.08-0.17)
*P* for trend	NA	.76	.21	NA	.74	.57
DASH						
1	106; 187	0.52 (0.45-0.60)	0.43 (0.35-0.51)	69; 194	0.17 (0.12-0.23)	0.11 (0.07-0.16)
2	93; 162	0.49 (0.41-0.57)	0.39 (0.31-0.47)	76; 202	0.10 (0.07-0.15)	0.08 (0.05-0.12)
3	124; 217	0.49 (0.43-0.56)	0.39 (0.33-0.47)	91; 219	0.16 (0.12-0.21)	0.12 (0.08-0.17)
4	127; 202	0.50 (0.43-0.57)	0.43 (0.36-0.51)	66; 189	0.12 (0.08-0.18)	0.10 (0.06-0.15)
*P* for trend	NA	.75	.90	NA	.33	.94
PBD						
1	95; 176	0.46 (0.38-0.53)	0.37 (0.30-0.45)	87; 209	0.15 (0.11-0.21)	0.09 (0.06-0.14)
2	119; 203	0.51 (0.44-0.58)	0.44 (0.37-0.51)	58; 167	0.14 (0.09-0.20)	0.10 (0.06-0.16)
3	136; 221	0.50 (0.43-0.57)	0.43 (0.36-0.50)	93; 222	0.15 (0.11-0.21)	0.12 (0.08-0.17)
4	100; 168	0.53 (0.45-0.61)	0.40 (0.32-0.48)	64; 206	0.11 (0.07-0.16)	0.09 (0.05-0.13)
*P* for trend	NA	.23	.80	NA	.28	.80

Abbreviations: AHA, American Heart Association; AHEI, Alternate Healthy Eating Index; AMD, alternate Mediterranean diet; DASH, Dietary Approach to Stop Hypertension; HEI, Healthy Eating Index; NA, not applicable; PBD, plant-based diet; PMD, Panagiotakos Mediterranean diet; TMD, Trichopoulou Mediterranean diet.

^a^ The model was adjusted for female age, body mass index, physical activity, and energy intake.

^b^ Analyses were run using generalized linear mixed models (proc glimmix) with random intercepts, binary distribution, and logit link.

^c^ Analyses were run using generalized linear mixed models (proc genmod) with random intercepts, binomial distribution, and logit link.

^d^ *P* < .05 for comparison of specific quartile vs quartile 1 (reference).

Nevertheless, adherence to the AHA dietary pattern was associated with a lower risk of total and clinical pregnancy loss among women who achieved a pregnancy during infertility treatment when combining data from IVF and IUI cycles (Figure; Table 3). In analyses combining information from IUI and IVF cycles, the adjusted probability of total pregnancy loss in the lowest quartile of the AHA pattern was 0.41 (95% CI, 0.33-0.50), whereas the adjusted probability in the highest quartile was 0.28 (95% CI, 0.21-0.36) (*P* for trend = .02). Similarly, the adjusted probability of clinical pregnancy loss in the lowest quartile was 0.30 (95% CI, 0.22-0.39), whereas the adjusted probability in the highest quartile was 0.15 (95% CI, 0.10-0.23) (*P* for trend = .007) (Figure). A similar pattern was observed for increasing DASH diet score, the 3 Mediterranean diet pattern scores, and the HEI and AHEI scores and risk of pregnancy loss, although these associations were weaker than that observed for the AHA diet pattern (Table 3). While the absolute risk difference of pregnancy loss comparing extreme quantiles of adherence for the AHA score was 15%, the same contrast for these other patterns ranged between 7% and 8%. Adherence to the PBD pattern was unrelated to pregnancy loss.

**Figure.**
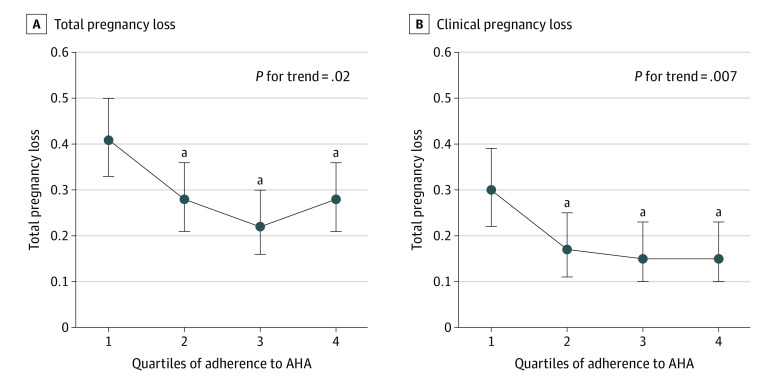
Association Between Adherence to the American Heart Association (AHA) Dietary Patterns and Total and Clinical Pregnancy Loss^a^ Association between female adherence to the AHA dietary patterns (according to quartiles of distribution) and the predicted probability of (A) total pregnancy loss and (B) clinical pregnancy loss following infertility treatment (intrauterine insemination or in vitro fertilization). Data are presented as predicted marginal proportions and 95% CIs. Analyses were run using generalized linear mixed models (proc glimmix) with random intercepts, binary distribution, and logit link. The model was adjusted for female age, body mass index, physical activity, and energy intake. ^a^*P* < .05 for comparison of specific quartile vs quartile 1 (reference).

**Table 3.** Association Between Women’s Adherence to the Different Dietary Patterns According to Quartiles of Distribution and the Predicted Probability of Total and Clinical Pregnancy Loss Following Infertility Treatment With Intrauterine Insemination or In Vitro Fertilization^a^

Quartile	Adjusted proportions (95% CI)	
	Total pregnancy loss	Clinical pregnancy loss
Cycles, No.	553	486
TMD		
1	0.33 (0.26-0.41)	0.24 (0.17-0.32)
2	0.29 (0.20-0.39)	0.19 (0.12-0.29)
3	0.32 (0.24-0.41)	0.17 (0.10-0.26)
4	0.25 (0.19-0.33)	0.17 (0.11-0.24)
*P* for trend	.21	.13
AMD		
1	0.35 (0.26-0.45)	0.24 (0.16-0.34)
2	0.32 (0.25-0.39)	0.23 (0.17-0.30)
3	0.26 (0.19-0.35)	0.14 (0.08-0.22)
4	0.27 (0.21-0.35)	0.17 (0.11-0.25)
*P* for trend	.19	.16
PMD		
1	0.37 (0.30-0.46)	0.26 (0.19-0.35)
2	0.30 (0.23-0.39)	0.18 (0.12-0.27)
3	0.25 (0.18-0.32)^b^	0.16 (0.11-0.24)
4	0.28 (0.21-0.36)	0.18 (0.12-0.27)
*P* for trend	.06	.13
HEI		
1	0.36 (0.29-0.45)	0.24 (0.17-0.33)
2	0.28 (0.20-0.36)	0.18 (0.11-0.26)
3	0.27 (0.20-0.35)	0.16 (0.11-0.24)
4	0.29 (0.22-0.37)	0.19 (0.13-0.27)
*P* for trend	.14	.29
AHEI		
1	0.34 (0.26-0.42)	0.24 (0.17-0.33)
2	0.30 (0.22-0.38)	0.19 (0.13-0.28)
3	0.27 (0.20-0.35)	0.19 (0.13-0.27)
4	0.29 (0.22-0.37)	0.16 (0.10-0.24)
*P* for trend	.30	.12
AHA index		
1	0.41 (0.33-0.50)	0.30 (0.22-0.39)
2	0.28 (0.21-0.36)^b^	0.17 (0.11-0.25)^b^
3	0.22 (0.16-0.30)^b^	0.15 (0.10-0.23)^b^
4	0.28 (0.21-0.36)^b^	0.15 (0.10-0.23)^b^
*P* for trend	.02	.007
DASH		
1	0.35 (0.27-0.43)	0.24 (0.17-0.32)
2	0.28 (0.20-0.38)	0.20 (0.13-0.30)
3	0.28 (0.22-0.36)	0.19 (0.13-0.27)
4	0.28 (0.21-0.35)	0.16 (0.10-0.23)
*P* for trend	.18	.13
PBD		
1	0.34 (0.27-0.42)	0.21 (0.14-0.29)
2	0.29 (0.22-0.39)	0.19 (0.12-0.28)
3	0.25 (0.19-0.33)	0.16 (0.10-0.23)
4	0.31 (0.23-0.40)	0.24 (0.16-0.33)
*P* for trend	.43	.69

Abbreviations: AHA, American Heart Association; AHEI, Alternate Healthy Eating Index; AMD, alternate Mediterranean diet; DASH, Dietary Approach to Stop Hypertension; HEI, Healthy Eating Index; PBD, plant-based diet; PMD, Panagiotakos Mediterranean diet; TMD, Trichopoulou Mediterranean diet.

^a^ The model was adjusted for female age, body mass index, physical activity, and energy intake. Analyses were run using generalized linear mixed models (proc glimmix) with random intercepts, binary distribution, and logit link. Total pregnancy loss was defined as β-hCG greater than 6 mIU/mL that did not result in live births (thus including biochemical and clinical pregnancy losses), and clinical pregnancy loss was defined as a clinical pregnancy that did not result in a live birth.

^b^ *P* < .05 for comparison of specific quartile vs quartile 1 (reference).

## Discussion

We evaluated the association between adherence to a priori–defined dietary patterns and infertility treatment outcomes among 612 women undergoing 1572 infertility treatment cycles. In partial support of our hypothesis, we found that adherence to the AHA diet, which prioritizes the consumption of fruits and vegetables, whole grains, fish and shellfish, and nuts and legumes while limiting the intake of sugar-sweetened beverages, sodium, processed meat, and saturated fats, was associated with a lower likelihood of pregnancy loss in women who achieved a pregnancy over the course of infertility treatment. A similar pattern was observed for all other dietary patterns, with the exception of the PBD dietary pattern. Nevertheless, none of the dietary patterns examined were associated with the probability of live birth.

To our knowledge, only 4 studies have examined the association between women’s pretreatment dietary patterns and infertility treatment outcomes to date,^10,11,28,29^ and the results across studies are inconsistent. Moreover, each study evaluated very few dietary patterns or only 1 at a time. For example, in the aforementioned articles, only 3 a priori healthy patterns were evaluated (PMD, TMD, and AHEI) in addition to 2 dietary patterns specifically developed for fertility (the Fertility Diet and the Profertility Diet). The present study expands the inquiry of the association between a priori–defined healthy patterns and 5 additional patterns (AMD, HEI, AHA, DASH, and PBD).

In a study of women undergoing infertility treatment in Greece, Karayiannis and colleagues^11^ reported a positive association between adherence to the PMD and live birth rates among 244 women without obesity undergoing their first IVF/ICSI treatment cycle. Our group subsequently reported no association between the AHEI dietary pattern with infertility treatment outcomes among 357 women undergoing ART in Massachusetts.^10^ In the same analysis, however, there was a suggestion of a positive association between adherence to PMD and live birth rates in a post hoc analysis. Moreover, a newly developed dietary pattern, the Profertility Diet, which prioritizes intake of supplemental folic acid, vitamin B12, vitamin D, low-pesticide fruits and vegetables, whole grains, seafood, dairy, and soy foods and limiting intake of high-pesticide fruits and vegetables, was associated with an increased probability of live birth among women undergoing ART.^10^ Briefly, in the nonvalidated Profertility Diet, participants receive 1 to 4 points based on increasing intake of the aforementioned profertility components and the scoring is reversed for intake of high-pesticide fruits and vegetables, resulting in a total score ranging from 9 to 36 points. The main differences in components between this score and the others detailed in this study are the consideration of vitamin D and vitamins of the 1-carbon metabolism (eg, vitamin B12 and B9). A third study evaluated the association between adherence to TMD and ART outcomes among 590 women undergoing infertility treatment in southern China.^29^ The authors reported that this pattern is associated with a larger number of embryos available for IVF but not pregnancy rates. In the most recent cohort study,^28^ the authors evaluated the association between adherence to the TMD pattern and IVF/ICSI outcomes among 474 subfertile couples undergoing infertility treatment in northern Italy and found no significant association with implantation, clinical pregnancy, or live birth. Finally, a randomized clinical trial aimed at evaluating the effect of adherence to a 6-week Mediterranean diet intervention for 6 weeks before IVF on embryo morphometric parameters among 111 women undergoing IVF in southern England found that the intervention altered embryo cleavage rate resulting in improved embryo quality.^12^ However, this trial was not powered to evaluate differences in clinical pregnancy or live birth rates.

It is important to consider the possible reasons underlying the discrepancies across studies. Although the dietary pattern scores evaluated in this study try to capture the same or very similar underlying information, the way in which the specific patterns are defined and scored is not identical (eTable 1 in Supplement 1). This is most evident for the 3 Mediterranean diet patterns, which are all aimed at describing the same dietary behavior yet approach this task differently. A consequence of this operational decision is that the distribution of specific foods (eTable 9 in Supplement 1) and nutrients (eTable 10 in Supplement 1) that may exert causal effects on a specific health outcome will differ across patterns. In this case, differences in intake between extreme quartiles of adherence for foods and nutrients previously related to infertility treatment outcomes, such as fish,^30,31^ whole grains,^32^ omega-3 fatty acids,^33^ and folic acid,^34^ were more pronounced for the AHA dietary pattern than for any other diet pattern examined (eTables 9 and 10 in Supplement 1). Of note, while the AHA dietary pattern exhibited significant differences in intake for these 4 dietary factors, the contrasts between extreme quartiles of adherence to AHA for these 4 factors were smaller than those previously associated with better infertility treatment outcomes.^30,34^ This also explains why dietary patterns specifically designed based on information for a specific outcome, such as the Profertility Diet, generally yield stronger associations with that specific outcome, as our group has previously reported.^10^ This information is nevertheless still useful, as it can be used in the design of dietary interventions, allowing investigators to favor intakes of specific foods and nutrients to maximize potential benefits, as some have done in studies aimed at testing the effect of diet patterns on fertility^12,35^ and chronic disease risk.^36^ Another important point to consider is that while it may be possible to assign scores across populations consistently, the same level of adherence to a specific dietary pattern may not reflect the same diet in Athens, Greece,^11^ Boston, Massachusetts,^10^ Guangdong Province, China,^29^ and Milan, Italy.^28^ Additional research aimed at identifying how overall dietary patterns and specific components of diet are related to human fertility is clearly needed, in particular research aimed at identifying dietary factors that can be replicated and implemented across cultural and culinary backgrounds.

### Limitations and Strengths

This study has limitations. First, this is a single-center study; therefore, the generalizability of our results is limited. Second, although our models were adjusted for several potential confounding factors, unmeasured confounding cannot be ruled out. Third, because of the observational nature of the study, our ability to determine causality is limited. Fourth, we only assessed diet at baseline, and therefore, we did not assess changes in diet over the study period. Fifth, preimplantation genetic testing for aneuploidy was not done routinely in these participants, so our models cannot account for euploidy. Sixth, it is unclear to what extent these findings are applicable to couples seeking pregnancy without medical assistance since the study population was composed of couples undergoing infertility treatment.

Despite these limitations, the study has several strengths. The main strength is the prospective design, reducing the possibility of reverse causality. Another strength includes, to our knowledge, the largest number of dietary patterns compared to date. An additional strength is the high level of standardization of the covariates, exposures, and outcomes of the study.

## Conclusions

In summary, our findings suggest that preconception adherence to the AHA diet pattern was inversely associated with total and clinical pregnancy loss in women who achieved pregnancy during the course of infertility treatment with IUI or IVF. A similar but weaker pattern was observed for all other dietary patterns except the PBD pattern. However, these differences were not sufficiently large to result in significant differences in the probability of achieving a live birth during the course of infertility treatment. The literature on the association between nutrition and infertility treatment outcomes remains sparse, and it is therefore important that this association is evaluated in additional studies. Nevertheless, our findings provide useful information that can be used in the design of future studies aimed at testing the effects of nutritional interventions on human fertility.

## Notes

Supplement 1. **eTable 1.** Components and Scoring Criteria for the Dietary Patterns Evaluated: Trichopoulou Mediterranean Diet (TMD),^1^ Alternate Mediterranean Diet (AMD),^2^ Panagiotakos Mediterranean Diet (PMD),^3^ Healthy Eating Index 2015 (HEI),^4^ Alternate Healthy Eating Index 2010 (AHEI),^5^ American Heart Association (AHA) Index,^6^ Dietary Approaches to Stop Hypertension 2005 (DASH) Index,^7^ and Plant-Based Diet Score (PBD)^8^ **eTable 2.** Baseline Demographic and Reproductive Characteristics of Study Participants Who Underwent IVF, Overall, and in the Lowest and Highest Quartiles of Women’s Adherence to the Different Dietary Patterns **eTable 3.** Baseline Demographic and Reproductive Characteristics of Study Participants Who Underwent IUI, Overall, and in the Lowest and Highest Quartiles of Women’s Adherence to the Different Dietary Patterns **eTable 4.** Baseline Demographic and Reproductive Characteristics of Study Participants Who Underwent IVF and IUI Cycles, Overall, and in the Lowest and Highest Quartiles of Women’s Adherence to the Different Dietary Patterns **eTable 5.** Association Between Women’s Adherence to A Priori–Defined Dietary Patterns According to Quartiles of Distribution and Probability of Clinical Pregnancy and Live Birth Following IVF or IUI Cycles **eTable 6.** Sensitivity Analysis: Association Between Women’s Adherence to A Priori–Defined Dietary Patterns According to Quartiles of Distribution and Probability of Clinical Pregnancy and Live Birth Following IVF After Exclusion of Canceled Cycles **eTable 7.** Sensitivity Analysis: Association Between Women’s Adherence to A Priori–Defined Dietary Patterns According to Quartiles of Distribution and Probability of Implantation, Clinical Pregnancy, and Live Birth Following Infertility Treatment (IVF Cycles) After Exclusion of Egg Donor Cycles **eTable 8.** Association Between Women’s Adherence to the Different Dietary Patterns According to Quartiles of Distribution and the Adjusted Means of Early Infertility Treatment Outcomes: Estradiol Trigger Levels and Endometrial Thickness **eTable 9.** Baseline Women’s Food Groups Information in the Lowest and Highest Quartiles of Women’s Adherence to the Different Dietary Patterns Following Infertility Treatment With IUI or IVF **eTable 10.** Baseline Women’s Reproductive Essential Micronutrients Intake Information in the Lowest and Highest Quartiles of Women’s Adherence to the Different Dietary Patterns Following Infertility Treatment With IUI or IVF **eFigure 1.** Flow Chart of the Participants Analyzed in the Present Study **eFigure 2.** Spearman Correlations Between Adherence to the Different Dietary Patterns in (A) IVF Cycles Only, (B) IUI Cycles Only, or (C) IUI Followed by IVF Cycles
eReferences.

Supplement 2. **Group Information. **EARTH Study Team

Supplement 3.
Data Sharing Statement
