# Supplementary material for: Women’s Adherence to Healthy Dietary Patterns and Outcomes of Infertility Treatment
Source: JAMA Netw Open. 2023 Aug 18;6(8):e2329982. doi: 10.1001/jamanetworkopen.2023.29982 (PMC10439476; doi:10.1001/jamanetworkopen.2023.29982)
Supplement: Supplement 1. — eTable 1. Components and Scoring Criteria for the Dietary Patterns Evaluated: Trichopoulou Mediterranean Diet (TMD),1 Alternate Mediterranean Diet (AMD),2 Panagiotakos Mediterranean Diet (PMD),3 Healthy Eating Index 2015 (HEI),4 Alternate Healthy Eating Index 2010 (AHEI),5 American Heart Association (AHA) Index,6 Dietary Approaches to Stop Hypertension 2005 (DASH) Index,7 and Plant-Based Diet Score (PBD)8 eTable 2. Baseline Demographic and Reproductive Characteristics of Study Participants Who Underwent IVF, Overall, and in the Lowest and Highest Quartiles of Women’s Adherence to the Different Dietary Patterns eTable 3. Baseline Demographic and Reproductive Characteristics of Study Participants Who Underwent IUI, Overall, and in the Lowest and Highest Quartiles of Women’s Adherence to the Different Dietary Patterns eTable 4. Baseline Demographic and Reproductive Characteristics of Study Participants Who Underwent IVF and IUI Cycles, Overall, and in the Lowest and Highest Quartiles of Women’s Adherence to the Different Dietary Patterns eTable 5. Association Between Women’s Adherence to A Priori–Defined Dietary Patterns According to Quartiles of Distribution and Probability of Clinical Pregnancy and Live Birth Following IVF or IUI Cycles eTable 6. Sensitivity Analysis: Association Between Women’s Adherence to A Priori–Defined Dietary Patterns According to Quartiles of Distribution and Probability of Clinical Pregnancy and Live Birth Following IVF After Exclusion of Canceled Cycles eTable 7. Sensitivity Analysis: Association Between Women’s Adherence to A Priori–Defined Dietary Patterns According to Quartiles of Distribution and Probability of Implantation, Clinical Pregnancy, and Live Birth Following Infertility Treatment (IVF Cycles) After Exclusion of Egg Donor Cycles eTable 8. Association Between Women’s Adherence to the Different Dietary Patterns According to Quartiles of Distribution and the Adjusted Means of Early Infertility Treatment Outcomes: Estradiol Trigge [file jamanetwopen-e2329982-s001.pdf]

Supplemental Online Content

Salas-Huetos A, Mitsunami M, Wang S, et al; EARTH Study Team. Women's adherence to healthy dietary patterns and outcomes of infertility treatment. *JAMA Netw Open*. 2023;6(8);e2329982. doi:10.1001/jamanetworkopen.2023.29982

eTable 1. Components and Scoring Criteria for the Dietary Patterns Evaluated: Trichopoulou Mediterranean Diet (TMD),[1] Alternate Mediterranean Diet (AMD),[2] Panagiotakos Mediterranean Diet (PMD),[3] Healthy Eating Index 2015 (HEI),[4] Alternate Healthy Eating Index 2010 (AHEI),[5] American Heart Association (AHA) Index,[6] Dietary Approaches to Stop Hypertension 2005 (DASH) Index,[7] and Plant-Based Diet Score (PBD)[8]

eTable 2. Baseline Demographic and Reproductive Characteristics of Study Participants Who Underwent IVF, Overall, and in the Lowest and Highest Quartiles of Women's Adherence to the Different Dietary Patterns

eTable 3. Baseline Demographic and Reproductive Characteristics of Study Participants Who Underwent IUI, Overall, and in the Lowest and Highest Quartiles of Women's Adherence to the Different Dietary Patterns

eTable 4. Baseline Demographic and Reproductive Characteristics of Study Participants Who Underwent IVF and IUI Cycles, Overall, and in the Lowest and Highest Quartiles of Women's Adherence to the Different Dietary Patterns

eTable 5. Association Between Women's Adherence to A Priori–Defined Dietary Patterns According to Quartiles of Distribution and Probability of Clinical Pregnancy and Live Birth Following IVF or IUI Cycles

eTable 6. Sensitivity Analysis: Association Between Women's Adherence to A Priori–Defined Dietary Patterns According to Quartiles of Distribution and Probability of Clinical Pregnancy and Live Birth Following IVF After Exclusion of Canceled Cycles

eTable 7. Sensitivity Analysis: Association Between Women's Adherence to A Priori–Defined Dietary Patterns According to Quartiles of Distribution and Probability of Implantation, Clinical Pregnancy, and Live Birth Following Infertility Treatment (IUI or IVF Cycles) After Exclusion of Egg Donor Cycles

eTable 8. Association Between Women's Adherence to the Different Dietary Patterns According to Quartiles of Distribution and the Adjusted Means of Early Infertility Treatment Outcomes: Estradiol Trigger Levels and Endometrial Thickness

© 2023 Salas-Huetos A et al. *JAMA Network Open*.

eTable 9. Baseline Women's Food Groups Information in the Lowest and Highest Quartiles of Women's Adherence to the Different Dietary Patterns Following Infertility Treatment With IUI or IVF

eTable 10. Baseline Women's Reproductive Essential Micronutrients Intake Information in the Lowest and Highest Quartiles of Women's Adherence to the Different Dietary Patterns Following Infertility Treatment With IUI or IVF

eFigure 1. Flow Chart of the Participants Analyzed in the Present Study

eFigure 2. Spearman Correlations Between Adherence to the Different Dietary Patterns in (A) IVF Cycles Only, (B) IUI Cycles Only, or (C) IUI Followed by IVF Cycles

eReferences.

This supplementary material has been provided by the authors to give readers additional information about their work.

© 2023 Salas-Huetos A et al. *JAMA Network Open*.

eTable 1. Components and Scoring Criteria for the Dietary Patterns Evaluated: Trichopoulou Mediterranean Diet (TMD),[1] Alternate Mediterranean Diet (AMD),[2] Panagiotakos Mediterranean Diet (PMD),[3] Healthy Eating Index 2015 (HEI),[4] Alternate Healthy Eating Index 2010 (AHEI),[5] American Heart Association (AHA) Index,[6] Dietary Approaches to Stop Hypertension 2005 (DASH) Index,[7] and Plant-Based Diet Score (PBD)[8]

Diet Pattern	Components	Definition/Clarification	Dietary recommendation	Standard for maximum points	Standard for minimum points
**TMD (maximum points=9)**	All grains	Refined and non-refined grains	Encourage consumption	>than median intake (servings/day) (1 point)	<than median intake (servings/day) (0 points)
	Fruits and nuts	Fruits, fruit juices and nuts (e.g., almonds, walnuts, etc.)	Encourage consumption	>than median intake (servings/day) (1 point)	<than median intake (servings/day) (0 points)
	Vegetables	Potatoes and French fries not included	Encourage consumption	>than median intake (servings/day) (1 point)	<than median intake (servings/day) (0 points)
	Legumes	Lentils, chickpeas, beans, etc.	Encourage consumption	>than median intake (servings/day) (1 point)	<than median intake (servings/day) (0 points)
	Fish	Fish and seafood	Encourage consumption	>than median intake (servings/day) (1 point)	<than median intake (servings/day) (0 points)
	Red meat, processed meat, and organ meats products	Read meat, processed meat, and offal (e.g., hamburger, hot dog, deli meat, beef, organs, etc., chicken not included)	Discourage consumption	>than median intake (servings/day) (0 point)	<than median intake (servings/day) (1 points)
	Dairy	All dairy products (e.g., full fat and low fat)	Discourage consumption	>than median intake (servings/day) (0 point)	<than median intake (servings/day) (1 points)
	Fats	MUFA/SFA	Encourage consumption	>than median intake (servings/day) (1 point)	<than median intake (servings/day) (0 points)
	Alcoholic beverages	Wine, beer, liquor, etc. 100 mL = 12 g ethanol (g/day)	Moderate consumption	Females:<br>5-25 g/day (1 point)<br>Males:<br>10-30 g/day (1 point)	Females:<br>0 point if ≤5 or ≥25 g/day (0 points)<br>Males:<br>≤10 or ≥30 g/day (0 points)
**AMD (maximum points=9)**	Whole grains	Whole grain (e.g., pasta, bread, etc.)	Encourage consumption	>than median intake (servings/day) (1 point)	<than median intake (servings/day) (0 points)
	Fruits	Fruits and fruit juices	Encourage consumption	>than median intake (servings/day) (1 point)	<than median intake (servings/day) (0 points)
	Vegetables	Potatoes and French fries not included	Encourage consumption	>than median intake (servings/day) (1 point)	<than median intake (servings/day) (0 points)
	Nuts	Nuts (e.g., almonds, walnuts, etc.)	Encourage consumption	>than median intake (servings/day) (1 point)	<than median intake (servings/day) (0 points)
	Legumes	Lentils, chickpeas, beans, etc.	Encourage consumption	>than median intake (servings/day) (1 point)	<than median intake (servings/day) (0 points)
	Fish	Fish and seafood	Encourage consumption	>than median intake (servings/day) (1 point)	<than median intake (servings/day) (0 points)
	Red meat, and processed meat	Read meat and processed meat (e.g., hamburger, hot dog, deli meat, beef, etc., chicken not included)	Discourage consumption	<than median intake (servings/day) (1 point)	>than median intake (servings/day) (0 points)
	Fats	MUFA/SFA	Encourage consumption	>than median intake (servings/day) (1 point)	<than median intake (servings/day) (0 points)

© 2023 Salas-Huetos A et al. *JAMA Network Open*.

	Alcoholic beverages	Wine, beer, liquor, etc. 100 mL = 12 g ethanol (g/day)	Moderate consumption	Females:<br>1 point if 5-15g/day (1 point)<br>Males:<br>1 point if 15-25g/day (1 point)	Females:<br>≤5 or ≥15g/day (0 points)<br>Males:<br>≤15 or ≥25g/day (0 points)
**PMD (maximum score 55)**	Whole grains	Whole grain (e.g., pasta, bread, etc.)	Encourage consumption	>32 servings/week (5 points)	No whole grains (0 points)
	Potatoes	French fries not included	Encourage consumption	>18 servings/week (5 points)	No potatoes (0 points)
	Fruits	Fruits and fruit juices	Encourage consumption	>22 servings/week (5 points)	No fruits (0 points)
	Vegetables	Potatoes and French fries not included	Encourage consumption	>33 servings/week (5 points)	No vegetables (0 points)
	Legumes	Lentils, chickpeas, beans, etc.	Encourage consumption	>6 servings/week (5 points)	No legumes (0 points)
	Fish	Fish and seafood	Encourage consumption	>6 servings/week (5 points)	No fish (0 points)
	Red meat, and processed meat	Read meat and processed meat (e.g., hamburger, hot dog, deli meat, beef, etc., chicken not included)	Discourage consumption	≤1 servings/week (5 points)	>10 (0 points)
	Poultry	Chicken	Discourage consumption	≤3 servings/week (5 points)	>10 (0 points)
	Full fat dairy products	All full fat dairy products (e.g., cheese, yoghurt, full fat milk, etc.)	Discourage consumption	≤10 servings/week (5 points)	>30 (0 points)
	Olive oil	Use of olive oil in cooking (times/week)	Encourage consumption	7 times/week (5 points)	Never (0 points)
	Alcoholic beverages	Wine, beer, liquor, etc. 100 mL = 12 g ethanol (mL/day)	Moderate consumption	<300 mL/day (5 points)	>700 mL/day or no alcohol (0 points)
**HEI (maximum points=100)**	Total fruit	Fruit and fruit juices	Encouraged	≥0.8 c equivalents/1000 kcal (5 points)	No fruit (0 points)
	Whole fruit	No fruit juices included	Encouraged	≥0.4 c equivalents/1000 kcal (5 points)	No whole fruit (0 points)
	Vegetables	Including green, yellow, cruciferous, etc.	Encouraged	≥1.1 c equivalents/1000 kcal (5 points)	No vegetables (0 points)
	Greens and beans	Legumes	Encouraged	≥0.2 c equivalents/1000 kcal (5 points)	No greens and beans (0 points)
	Whole grains	Including pasta, bread, etc.	Encouraged	≥1.5 oz equivalents/1000 kcal (10 points)	No whole grains (0 points)
	Dairy	Including low and high fat dairy	Encouraged	≥1.3 c equivalents/1000 kcal (10 points)	No dairy (0 points)
	Total protein foods	Including read meat, processed meat, fish, poultry, legumes etc.	Encouraged	≥2.5 oz equivalents/1000 kcal (5 points)	No protein foods (0 points)
	Seafood and plant proteins	Including fish, shellfish, nuts, legumes, soy products, etc.	Encouraged	≥0.8 oz equivalents/1000 kcal (5 points)	No seafood or plant proteins (0 points)
	Fatty acids	Ratio of PUFAs and MUFAs fatty acids to SFAs	Encouraged	(PUFA+MUFA)/SFA≥2.5 (10 points)	(PUFA+MUFA)/SFA≤1.2 (0 points)

© 2023 Salas-Huetos A et al. *JAMA Network Open*.

	Refined grains	Including white flour, white rice, white bread, etc.	Discouraged	≤1.8 oz equivalents/1000 kcal (10 points)	≥4.3 oz equivalents/1000 kcal (0 points)
	Sodium	Sodium intake	Discouraged	≤1.1 g/1000 kcal (10 points)	≥2.0 g/1000 kcal (0 points)
	Added sugars	Added sugars intake	Discouraged	≤6.5% energy/1000 kcal (10 points)	≥26% energy/1000 kcal (0 points)
	Saturated fats	Saturated fats intake	Discouraged	≤8% energy/1000 kcal (10 points)	≥16% energy/1000 kcal (0 points)
**AHEI (maximum points=100)**	Fruit	No fruit juices included	Encouraged	≥4 servings/day (10 points)	No vegetables (0 points)
	Vegetables	Including green, yellow, cruciferous, etc.	Encouraged	≥5 servings/day (10 points)	No fruit (0 points)
	Nuts and legumes	Nuts and legumes consumption	Encouraged	≥1 servings/day (10 points)	No nuts and legumes (0 points)
	Whole grains	Including pasta, bread, etc.	Encouraged	Women: ≥75 g/day (10 points)<br>Men: ≥90 g/day (10 points)	No whole grains (0 points)
	Sugar-sweetened beverages	Including high energy drinks and fruit juices	Discouraged	No sugar-sweetened beverages (10 points)	≥8 oz/day (0 points)
	Red and processed meat	Including read meat, and processed meat	Discouraged	No red and processed meat (10 points)	≥1.5 servings/day (0 points)
	Long chain omega-3 fats (EPA+DHA)	EPA+DHA intake	Encouraged	≥ 250 mg/day (10 points)	No EPA+DHA intake (0 points)
	PUFA	PUFAs intake	Encouraged	≥ 10% energy (10 points)	≤ 2% energy (0 points)
	Alcohol	Alcohol intake	Discouraged (moderate intake)	Women: 0.5-1.5 drinks/day (10 points)<br>Men: 0.5-2.0 drinks/day (10 points)	Women: ≥2.5 drinks/day (0 points)<br>Men: ≥3.5 drinks/day (0 points)
	Sodium	Sodium intake	Discouraged	Lowest decile (10 points)	Highest decile (0 points)
**AHA (maximum points=80)**	Fruits and vegetables	No fruit juices included	Encouraged	≥4.5 cups/day (10 points)	0 cups/day (0 points)
	Whole grain	Including pasta, bread, etc.	Encouraged	≥3.0 oz-equivalents/day (10 points)	0 oz-equivalents/day (0 points)
	Fish and shellfish	No bread fish included	Encouraged	≥2.0 servings/week (10 points)	0 servings/week (0 points)
	Sugar-sweetened beverages	Including high energy drinks and fruit juices	Discouraged	≤5.1 fluid oz/day (10 points)	>16 fluid oz/day (0 points)
	Sodium	Sodium intake (energy adjusted)	Discouraged	≤1.5 grams/day (10 points)	>4.5 energy adjusted grams/day (0 points)
	Nuts, seeds, and legumes	No soy products included	Encouraged	≥1.0 servings/day (10 points)	0 servings/day (0 points)
	Processed meat	No read meat included	Discouraged	≤0.5 oz/day (10 points)	>1.8 oz/day (0 points)
	Saturated fat	Only SFAs included	Discouraged	≤7.0% of total calories/day (10 points)	>15% of total calories/day (0 points)
**DASH (maximum points=40)**	Total fruit	Fruit and fruit juices	Encouraged	≥5.0 servings/day (5 points)	0 servings/day (1 point)
	Vegetables	No legumes and soy products included	Encouraged	≥2.6 servings/day (5 points)	0 servings/day (1 point)
	Nuts and legumes	Including legumes, nuts, and soy products	Encouraged	≥0.4 servings/day (5 points)	0 servings/day (1 point)
	Whole grain	Including pasta, bread, etc.	Encouraged	≥5.0 servings/day (5 points)	0 servings/day (1 point)
	Low-fat dairy	No high-fat dairy included	Encouraged	≥5.0 servings/day (5 points)	0 servings/day (1 point)

© 2023 Salas-Huetos A et al. *JAMA Network Open*.

	Red and processed meat	Including read meat, and processed meat	Discouraged	≤0.3 servings/day (5 points)	>2.1 servings/day (1 point)
	Sugar-sweetened beverages	Including high energy and carbonated drinks, and punch beverages	Discouraged	≤0.1 servings/day (5 points)	>3.0 servings/day (1 point)
	Sodium	Sodium intake	Discouraged	≤2.6 servings/day (5 points)	>2.7 servings/day (1 point)
**PBD (maximum points=60)**	Vegetables	No legumes and soy products included	Encouraged	≥2 servings/d (5 points)	<2 servings/d (1 point)
	Total fruit	Fruit and fruit juices	Encouraged	≥ 3 servings/d (5 points)	< 3 servings/d (1 point)
	Legumes	Legumes	Encouraged	≥ 3 servings/week (5 points)	< 3 servings/week (1 point)
	All grain	Refined and non-refined grains	Encouraged	≥ 2 servings/d (5 points)	< 2 servings/d (1 point)
	Potatoes	Potatoes and chips	Encouraged	≥ 1 servings/d (5 points)	< 1 servings/d (1 point)
	Nuts	Nuts (e.g., almonds, walnuts, etc.)	Encouraged	≥ 3 servings/week (5 points)	< 3 servings/week (1 point)
	Olive oil	Refined, extra-virgin, etc.	Encouraged	≥ 4 tablespoons/d (5 points)	< 4 tablespoons/d (1 point)
	All meat and meat products	Red meat, meat, poultry, organs, etc.	Discouraged	≤3 servings/week (5 points)	>3 servings/week (1 point)
	Animal fats	For cooking or as a spread (butter)	Discouraged	≤3 servings/week (5 points)	>3 servings/week (1 point)
	Eggs	Egg consumption	Discouraged	≤3 servings/week (5 points)	>3 servings/week (1 point)
	Fish and seafood	White fish, blue fish, mussels, shrimp, etc.	Discouraged	≤3 servings/week (5 points)	>3 servings/week (1 point)
	Dairy products	High-fat and low-fat dairy	Discouraged	≤1 servings/day (5 points)	>1 servings/day (1 point)

Abbreviations: AHA, American Heart Association Index; AHEI, Alternate Healthy Eating Index; AMD, Alternate Mediterranean diet; DASH, Dietary Approach to Stop Hypertension index; DHA, docosahexaenoic omega-3 fatty acid; EPA, eicosapentaenoic omega-3 fatty acid; HEI, Healthy Eating Index; MUFA, monounsaturated fatty acids; PBD, Plant-based diet; PMD, Panagiotakos Mediterranean diet; PUFA, polyunsaturated fatty acids; SFA, saturated fatty acids; TMD, Trichopoulou Mediterranean diet.

© 2023 Salas-Huetos A et al. *JAMA Network Open*.

eTable 2. Baseline Demographic and Reproductive Characteristics of Study Participants Who Underwent IVF, Overall, and in the Lowest and Highest Quartiles of Women's Adherence to the Different Dietary Patterns

	Overall	TMD		AMD		PMD		HEI		AHEI		AHA		DASH		PBD	
		Q1 (0-3)	Q4 (6-9)	Q1 (0-2)	Q4 (6-9)	Q1 (17-26)	Q4 (33-43)	Q1 (43.8-60.6)	Q4 (72.8-86.1)	Q1 (23-52)	Q4 (66-89)	Q1 (15-41)	Q4 (58-79)	Q1 (13-20)	Q4 (27-36)	Q1 (22-31)	Q4 (40-51)
n	450	141	141	86	128	104	110	107	120	112	116	108	108	106	127	95	100
Female demographic characteristics																	
Age (y), median (IQR)	35.0 (32.0, 39.0)	35.0 (31.0, 38.0)	35.0 (33.0, 38.0)	34.5 (31.0, 37.0)	35.5 (33.0, 39.0)	35.5 (32.0, 39.0)	34.0 (33.0, 39.0)	34.0 (32.0, 37.0)	36.0 (33.0, 40.0) [a]	34.0 (31.5, 38.0)	35.0 (32.0, 38.0)	34.0 (32.0, 37.0)	35.0 (33.0, 39.0)	35.0 (32.0, 38.0)	35.0 (32.0, 39.0) [a]	36.0 (32.0, 39.0)	35.0 (33.0, 38.0)
BMI (kg/m^2), median (IQR)	23.2 (21.2, 26.0)	23.1 (21.6, 25.7)	23.1 (21.0, 26.0)	23.1 (21.2, 25.7)	23.3 (21.2, 26.0)	23.3 (21.2, 25.8)	22.1 (20.6, 25.0) [a]	23.2 (20.9, 26.0)	22.7 (21.0, 25.7)	23.2 (21.2, 25.8)	23.2 (21.2, 25.0)	24.2 (22.1, 27.1)	22.5 (21.0, 25.0) [a]	23.3 (21.2, 25.9)	22.1 (20.8, 24.7) [a]	23.1 (21.2, 26.0)	22.4 (20.6, 26.3)
Race, white, n (%)	372 (83.0)	117 (83.6)	113 (80.7)	68 (80.0)	110 (86.6)	83 (79.8)	88 (80.7)	93 (86.9)	96 (80.7)	91 (82.0)	99 (86.1)	97 (90.7)	81 (75.7) [a]	88 (83.0)	103 (81.8)	77 (81.9)	79 (79.8)
Smoking status, ever smoker, n (%)	107 (23.8)	31 (22.1)	36 (25.5)	21 (24.7)	30 (23.4)	30 (28.9)	24 (21.8)	27 (25.2)	24 (20.0)	32 (28.8)	28 (24.1)	30 (28.0)	22 (20.4)	29 (27.4)	31 (24.4)	30 (31.9)	25 (25.0)
Education, college or higher, n (%)	398 (92.3)	125 (94.7)	127 (92.7)	71 (91.0)	116 (92.8)	88 (91.7)	103 (97.2)	89 (90.8)	107 (91.5)	91 (86.7)	105 (93.8) [a]	92 (91.2)	99 (94.3)	88 (90.7)	110 (90.2)	78 (90.7)	88 (91.7)
Moderate-to-vigorous physical activity (min/week) [b], median (IQR)	128.5 (29.5, 300.0)	95.0 (5.0, 281.0)	174.0 (83.0, 390.0) [a]	60.0 (0.0, 240.0)	179.8 (83.5, 389.8) [a]	60.0 (0.0, 211.3)	179.5 (71.0, 390.0) [a]	90 (2.5, 240.0)	179.5 (65.5, 390.0) [a]	90.0 (1.3, 225.0)	161.5 (90.0, 335.8) [a]	66.0 (3.8, 197.3)	155.0 (68.5, 319.8) [a]	89.8 (2.5, 210.0)	179.5 (72.0, 390.0) [a]	84.0 (2.5, 300.0)	156.0 (66.0, 335.0)
Female energy intake (kcal/day), median (IQR)	1687 (1354, 2086)	1446 (1150, 1709)	1962 (1629, 2358) [a]	1462 (1086, 1739)	1965 (1646, 2347) [a]	1562 (1214, 1859)	1994 (1627, 2351) [a]	1682 (1390, 2014)	1751 (1405, 2241) [a]	1610 (1269, 1974)	1783 (1477, 2205)	1560 (1214, 1891)	1849 (1594, 2217) [a]	1560 (1187, 1919)	1817 (1505, 2219) [a]	1498 (1212, 1793)	1881 (1530, 2398) [a]
Multivitamin use (tablets/week), median (IQR)	3 (3, 3)	3 (3, 3)	3 (3, 3)	3 (2, 3)	3 (3, 3)	3 (3, 3)	3 (3, 3)	3 (2, 3)	3 (3, 3)	3 (2, 3)	3 (3, 3)	3 (3, 3)	3 (3, 3)	3 (2, 3)	3 (3, 3)	3 (2, 3)	3 (3, 3)
Couple cycle characteristics																	
Infertility diagnosis, n (%)		NA	S	NA	NS	NA	NS	NA	NS	NA	NS	NA	NS	NA	NS	NA	NS
Female factor	153 (34.0)	52 (36.9)	61 (43.3)	36 (41.9)	53 (41.4)	44 (42.3)	48 (43.6)	43 (40.2)	47 (39.2)	41 (36.6)	46 (39.7)	40 (37.0)	47 (43.5)	39 (36.8)	49 (38.6)	41 (43.2)	43 (43.0)
Male factor	153 (34.0)	45 (31.9)	41 (29.1)	25 (29.1)	37 (28.9)	36 (34.6)	33 (30.0)	38 (35.5)	35 (29.2)	38 (33.9)	34 (29.3)	35 (32.4)	34 (31.5)	34 (32.1)	42 (33.1)	28 (29.5)	31 (31.0)
Unexplained	124 (27.6)	44 (31.2)	39 (27.7)	25 (29.1)	38 (29.7)	24 (23.1)	29 (26.4)	26 (24.3)	38 (31.7)	33 (29.5)	36 (31.0)	33 (30.6)	27 (25.0)	33 (31.1)	36 (28.4)	26 (27.4)	26 (26.0)
Treatment protocol, n (%)		NA	NS	NA	NS	NA	NS	NA	NS	NA	NS	NA	NS	NA	NS	NA	NS
Antagonist	68 (15.1)	16 (11.4)	25 (17.7)	6 (7.0)	18 (14.1)	15 (14.4)	20 (18.2)	15 (14.0)	24 (20.0)	12 (10.7)	24 (20.7)	13 (12.0)	17 (15.7)	12 (11.3)	21 (16.5)	17 (17.9)	17 (17.0)
Flare [c]	50 (11.1)	14 (9.9)	13 (9.2)	10 (11.6)	14 (10.9)	13 (12.5)	6 (5.5)	9 (8.4)	14 (11.7)	10 (8.9)	10 (8.6)	9 (8.3)	12 (11.1)	14 (13.2)	9 (7.1)	13 (13.7)	10 (10.0)
Luteal phase agonist [d]	283 (62.9)	91 (64.5)	91 (64.5)	59 (68.6)	85 (66.4)	61 (58.7)	74 (67.3)	72 (67.3)	70 (58.3)	77 (68.8)	72 (62.1)	74 (68.5)	66 (61.1)	69 (65.1)	83 (65.4)	55 (57.9)	61 (61.0)
Egg donor or cryo cycle	49 (10.9)	20 (14.2)	12 (8.5)	11 (12.8)	11 (8.6)	15 (14.4)	10 (9.1)	11 (10.3)	12 (10.0)	13 (11.6)	10 (8.6)	12 (11.1)	13 (12.0)	11 (10.4)	14 (11.0)	10 (10.5)	12 (12.0)

© 2023 Salas-Huetos A et al. *JAMA Network Open*.

Embryo transfer day, n (%)		NA	NS	NA	NS	NA	NS	NA	S	NA	NS	NA	NS	NA	NS	NA	NS
Day 2	16 (4.6)	4 (3.8)	8 (7.0)	2 (3.1)	5 (4.9)	4 (5.2)	6 (6.7)	4 (4.7)	9 (9.7)	4 (4.6)	7 (7.6)	4 (4.8)	8 (9.3)	3 (3.7)	8 (8.1)	1 (1.4)	7 (9.7)
Day 3	161 (46.7)	40 (37.7)	53 (46.5)	24 (36.9)	54 (52.9)	32 (41.6)	42 (47.2)	37 (43.5)	50 (53.8)	35 (40.2)	42 (45.7)	37 (44.1)	42 (48.8)	35 (42.7)	50 (50.5)	37 (50.0)	38 (52.8)
Day 5	168 (48.7)	62 (58.5)	53 (46.5)	39 (60.0)	43 (42.2)	41 (53.3)	41 (46.1)	44 (51.8)	34 (36.6)	48 (55.2)	43 (46.7)	43 (51.2)	36 (41.9)	44 (53.7)	41 (41.4)	36 (48.7)	27 (37.5)
Number of embryos transferred, n (%)		NA	NS	NA	NS	NA	NS	NA	S	NA	NS	NA	NS	NA	NS	NA	NS
One embryo	100 (29.1)	36 (34.0)	30 (26.3)	24 (36.9)	32 (31.4)	25 (32.5)	28 (31.5)	24 (28.2)	23 (24.7)	25 (28.7)	30 (32.6)	23 (27.4)	29 (34.1)	20 (24.4)	32 (32.3)	17 (23.0)	22 (30.6)
Two embryos	183 (52.9)	52 (49.1)	66 (57.9)	33 (50.8)	50 (49.0)	38 (49.4)	46 (51.7)	45 (52.9)	43 (46.2)	52 (59.8)	44 (47.8)	51 (60.7)	39 (45.9)	49 (59.8)	46 (46.5)	46 (62.2)	39 (54.2)
Three or more embryos	62 (18.0)	18 (17.0)	18 (15.8)	8 (12.3)	20 (19.6)	14 (18.2)	15 (16.9)	16 (18.8)	27 (29.0)	10 (11.5)	18 (19.6)	10 (11.9)	17 (20.0)	13 (15.9)	21 (21.2)	11 (14.9)	11 (15.3)
Male demographic characteristics																	
Age (y), median (IQR)	36.5 (32.9, 40.0)	34.7 (32.4, 38.4)	36.8 (33.3, 40.4) [a]	34.6 (32.5, 38.4)	36.2 (32.5, 41.0)	37.6 (32.9, 39.6)	36.6 (33.6, 41.1)	35.0 (32.4, 39.4)	37.4 (34.5, 41.0)	35.9 (32.7, 38.8)	35.5 (33.6, 39.5)	34.7 (32.6, 38.5)	36.8(33.6, 39.9)	35.7 (32.1, 39.4)	36.4 (32.6, 39.3) [a]	35.7 (33.0, 40.4)	36.4 (32.6, 40.1)
BMI (kg/m^2), median (IQR)	26.9 (24.3, 29.3)	27.0 (24.4, 29.1)	26.4 (23.5, 28.7)	27.1 (24.7, 28.8)	26.6 (23.7, 30.0)	27.8 (25.4, 30.2)	26.5 (23.8, 28.0)	27.5 (25.4, 30.0)	26.3 (23.7, 29.1)	27.9 (25.4, 29.9)	26.2 (23.6, 29.7)	27.6 (25.4, 29.8)	26.9 (24.5, 28.9)	27.4 (24.6, 29.8)	26.5 (23.8, 30.0)	26.9 (24.3, 29.1)	26.4 (24.4, 29.1)
Race, white, n (%)	240 (88.9)	83 (89.3)	66 (91.7)	59 (89.4)	61 (89.7)	52 (91.3)	71 (87.7)	63 (88.7)	58 (90.6)	73 (89.0)	65 (95.6)	66 (93.0)	54 (87.1)	57 (85.1)	60 (87.0)	50 (89.3)	47 (82.5)
Smoking status, ever smoker, n (%)	86 (31.7)	31 (33.3)	22 (30.1)	22 (33.3)	18 (26.1)	20 (35.1)	23 (28.1)	21 (29.6)	19 (29.2)	29 (35.4)	21 (30.4)	24 (33.8)	15 (23.8)	20 (29.9)	18 (25.7)	21 (37.5)	11 (19.0)
Education, college or higher, n (%)	188 (85.1)	67 (83.8)	54 (91.5)	46 (82.1)	46 (92.0)	38 (82.6)	61 (92.4)	49 (83.1)	41 (83.7)	54 (79.4)	46 (88.5)	49 (83.1)	43 (91.5)	43 (81.1)	46 (83.6)	40 (85.1)	39 (84.8)
Moderate-to-vigorous physical activity (min/week) [b], median (IQR)	150.0 (0.0, 372.0)	150.0 (24.0, 390.0)	180.0 (41.5, 390.0)	144.8 (0.0, 389.5)	161.0 (0.0, 390.0)	41.5 (0.0, 164.0)	180.0 (29.5, 390.0) [a]	90.0 (0.0, 359.5)	132.0 (0.0, 372.0)	108.0 (0.0, 300.0)	150.0 (0.0, 346.0)	114.0 (2.5, 300.0)	148.5 (0.0, 359.0)	90.0 (0.0, 431.5)	119.5 (0.0, 332.5)	145.0 (23.3, 434.8)	149.8 (5.0, 372.0)

Data are presented as median (interquartile range) for continuous variables or n (%) for categorical variables. P-values were calculated using a Kruskal-Wallis test for continuous variables and a Chi-square test for categorical variables.

[a] P<0.05 for comparison of quartile 4 versus quartile 1 (reference). [b] Includes weight and aerobic exercise and sports. [c] Follicular-phase GnRH agonist/flare protocol. [d] Luteal-phase GnRH agonist protocol.

Abbreviations: AHA, American Heart Association diet recommendations; AHEI, Alternate Healthy Eating Index; AMD, Alternate Mediterranean diet; ART, assisted reproductive technologies; BMI, body mass index; DASH, Dietary Approaches to Stop Hypertension diet; HEI, Healthy Eating Index; IQR, interquartile range; n, sample size; NA, not applicable; NS, non-statistically significant (P-value>0.05); PBD, Plant-based diet; PMD, Panagiotakos Mediterranean diet; Q, quartile; S, statistically significant (P-value<0.05); TMD, Trichopoulou Mediterranean diet.

© 2023 Salas-Huetos A et al. *JAMA Network Open*.

eTable 3. Baseline Demographic and Reproductive Characteristics of Study Participants Who Underwent IUI, Overall, and in the Lowest and Highest Quartiles of Women's Adherence to the Different Dietary Patterns

	Overall	TMD		AMD		PMD		HEI		AHEI		AHA		DASH		PBD	
		Q1 (0-3)	Q4 (7-9)	Q1 (0-2)	Q4 (6-9)	Q1 (17-26)	Q4 (33-44)	Q1 (43.0-60.0)	Q4 (72.6-86.7)	Q1 (28-50)	Q4 (66-87)	Q1 (15-40)	Q4 (59-76)	Q1 (13-19)	Q4 (28-35)	Q1 (23-32)	Q4 (40-56)
n	302	91	48	56	91	78	76	68	81	67	80	68	72	69	66	87	64
Female demographic characteristics																	
Age (y), median (IQR)	34.0 (32.0, 37.5)	34.0 (31.0, 37.0)	35.0 (32.0, 37.5)	34.0 (30.0, 36.0)	34.0 (32.0, 38.0)	35.0 (32.0, 38.0)	35.0 (32.0, 38.0)	33.5 (30.0, 36.0)	36.0 (33.0, 38.0)	33.0 (31.0, 36.0)	35.0 (32.0, 38.0) [a]	34.0 (30.0, 37.0)	35.0 (32.5, 38.0)	34.0 (32.0, 38.0)	34.0 (32.0, 38.0) [a]	34.0 (31.0, 38.0)	34.5 (32.0, 38.0)
BMI (kg/m^2), median (IQR)	23.7 (21.3, 26.6)	24.8 (22.4, 27.6)	22.6 (21.2, 26.4)	24.8 (22.9, 27.5)	23.6 (21.2, 27.3)	24.9 (22.4, 27.0)	22.7 (21.2, 27.4)	24.0 (21.8, 26.4)	22.8 (21.1, 26.4)	24.0 (21.6, 26.7)	22.9 (21.2, 26.4)	24.3 (22.4, 27.0)	22.7 (21.0, 26.2)	24.7 (22.0, 26.7)	22.7 (20.4, 26.4)	24.8 (22.5, 27.2)	22.8 (21.0, 26.7)
Race, white, n (%)	244 (81.3)	70 (77.8)	36 (76.6)	42 (76.4)	76 (84.4)	63 (80.8)	60 (80.0)	58 (85.3)	65 (81.3)	58 (86.6)	64 (81.0)	61 (91.0)	52 (73.2)	55 (79.7)	50 (76.9)	67 (77.9)	49 (77.8)
Smoking status, ever smoker, n (%)	81 (26.9)	15 (16.7)	13 (27.1)	11 (20.0)	26 (28.6)	24 (30.8)	17 (22.4)	17 (25.0)	22 (27.2)	16 (23.9)	24 (30.0)	18 (26.9)	22 (30.6)	21 (30.4)	20 (30.3)	26 (30.2)	16 (25.0)
Education, college or higher, n (%)	269 (93.4)	80 (93.0)	47 (100.0) [a]	48 (90.6)	90 (100.0) [a]	64 (86.5)	75 (100.0) [a]	54 (85.7)	77 (96.3) [a]	58 (89.2)	77 (98.7)	56 (87.5)	70 (98.6) [a]	55 (84.6)	61 (95.3) [a]	70 (89.7)	58 (93.6)
Moderate-to-vigorous physical activity (min/week) [b], median (IQR)	150.0 (41.5, 314.5)	149.0 (19.5, 341.5)	221.8 (90.0, 390.0)	149.3 (21.8, 350.3)	179.5 (84.0, 359.0)	119.8 (12.0, 300.0)	168.0 (71.5, 380.3)	111.8 (26.8, 290.5)	174.0 (72.0, 390.0)	120.0 (29.5, 332.5)	179.5 (76.0, 352.8)	95.8 (21.8, 290.5)	176.8 (72.8, 374.5)	114.0 (29.5, 240.0)	158.0 (60.0, 329.5)	120.0 (2.5, 362.5)	161.8 (75.3, 344.3)
Female energy intake (kcal/day), median (IQR)	1646 (1356, 2037)	1417 (1190, 1703)	1893 (1683, 2277) [a]	1378 (1144, 1657)	1911 (1615, 2278) [a]	1558 (1257, 1869)	1936 (1715, 2312) [a]	1601 (1355, 2036)	1752 (1538, 2142) [a]	1555 (1225, 1941)	1803 (1563, 2130) [a]	1518 (1205, 1891)	1808 (1590, 2147) [a]	1537 (1198, 1888)	1781 (1486, 2049) [a]	1538 (1212, 1864)	1895 (1598, 2179) [a]
Multivitamin use (tablets/week), median (IQR)	3 (2, 3)	3 (2, 3)	3 (2, 3)	3 (2, 3)	3 (2, 3)	3 (2, 3)	3 (2, 3)	3 (2, 3)	3 (3, 3)	3 (2, 3)	3 (3, 3)	3 (2, 3)	3 (3, 3)	3 (2, 3)	3 (3, 3)	3 (2, 3)	3 (2, 3) [a]
Couple cycle characteristics																	
Infertility diagnosis, n (%)		NA	NS	NA	NS	NA	S	NA	NS	NA	NS	NA	NS	NA	NS	NA	S
Female factor	48 (16.2)	15 (17.1)	4 (8.5)	8 (15.1)	10 (11.1)	10 (13.0)	7 (9.2)	12 (17.9)	9 (11.3)	10 (15.4)	9 (11.4)	12 (18.2)	10 (14.1)	12 (17.7)	13 (19.7)	7 (8.2)	7 (10.9)
Male factor	125 (42.1)	38 (43.2)	24 (51.1)	23 (43.4)	40 (44.4)	38 (49.4)	39 (51.3)	33 (49.3)	31 (38.8)	30 (46.2)	36 (45.6)	30 (45.5)	32 (45.1)	29 (42.7)	26 (39.4)	41 (48.2)	33 (51.6)
Unexplained	124 (41.8)	35 (39.8)	19 (40.4)	22 (41.5)	40 (44.4)	29 (37.7)	30 (39.5)	22 (32.8)	40 (50.0)	25 (38.5)	34 (43.0)	24 (36.4)	29 (40.9)	27 (39.7)	27 (40.9)	37 (43.5)	24 (37.5)
Male demographic characteristics																	
Age (y), median (IQR)	35.2 (32.4, 38.8)	33.8 (31.2, 37.7)	38.2 (33.2, 41.9) [a]	33.6 (31.7, 35.4)	36.8 (33.0, 40.9) [a]	33.9 (31.9, 38.1)	36.8 (33.3, 40.5)	33.8 (31.7, 38.4)	38.3 (34.5, 41.5) [a]	34.1 (31.7, 38.4)	36.5 (33.9, 40.2)	33.8 (31.3, 38.1)	37.4 (33.9, 39.0)	33.6 (30.5, 37.8)	37.4 (33.9, 40.2) [a]	34.3 (31.2, 38.4)	38.0 (33.0, 41.5)
BMI (kg/m^2), median (IQR)	27.8 (24.7, 30.8)	28.5 (27.3, 31.1)	26.9 (24.9, 29.8) [a]	28.1 (25.4, 31.0)	27.4 (24.9, 30.7)	28.4 (25.4, 31.1)	27.1 (24.9, 30.5)	28.5 (27.3, 31.1)	27.3 (24.7, 30.5)	28.1 (24.9, 30.0)	27.3 (24.9, 30.6)	29.3 (27.3, 31.2)	28.1 (25.1, 30.5)	27.8 (25.0, 31.0)	25.4 (24.1, 29.4)	27.9 (25.0, 30.8)	25.6 (24.0, 28.1)
Race, white, n (%)	134 (90.5)	43 (89.6)	21 (87.5)	30 (88.2)	44 (89.8)	30 (90.9)	48 (90.6)	37 (92.5)	34 (89.5)	37 (92.5)	36 (92.3)	36 (97.3)	32 (86.5)	31 (91.2)	31 (91.2)	37 (90.2)	26 (83.9)

© 2023 Salas-Huetos A et al. *JAMA Network Open*.

Smoking status, ever smoker, n (%)	49 (33.1)	18 (37.5)	7 (29.2)	14 (41.2)	16 (32.7)	13 (39.4)	19 (35.9)	16 (40.0)	13 (34.2)	14 (35.0)	13 (33.3)	14 (37.8)	11 (29.7)	11 (32.4)	10 (29.4)	13 (31.7)	7 (22.6)
Education, college or higher, n (%)	110 (88.0)	34 (85.0)	19 (95.0)	26 (89.7)	38 (90.5)	22 (84.6)	43 (89.6)	26 (86.7)	30 (90.9)	25 (78.1)	30 (88.2)	27 (93.1)	30 (93.8)	24 (88.9)	27 (90.0)	31 (88.6)	25 (89.3)
Moderate-to-vigorous physical activity (min/week) [b], median (IQR)	150.0 (12.0, 371.5)	130.5 (0.0, 326.8)	97.8 (48.0, 344.0)	142.0 (0.0, 389.5)	164.5 (37.0, 400.5)	150.0 (0.0, 431.5)	179.5 (59.0, 400.5)	142.0 (0.0, 311.5)	119.0 (60.0, 316.5)	150.0 (0.0, 371.5)	150.0 (60.0, 390.0)	150.0 (0.0, 360.0)	89.5 (37.0, 359.0)	150.0 (0.0, 431.5)	88.0 (37.0, 240.0)	214.0 (29.5, 390.0)	180.0 (22.0, 420.0)

Data are presented as median (interquartile range) for continuous variables or n (%) for categorical variables. P-values were calculated using a Kruskal-Wallis test for continuous variables and a Chi-square test for categorical variables.

[a] P<0.05 for comparison of quartile 4 versus quartile 1 (reference). [b] Includes weight and aerobic exercise and sports.

Abbreviations: AHA, American Heart Association diet recommendations; AHEI, Alternate Healthy Eating Index; AMD, Alternate Mediterranean diet; ART, assisted reproductive technologies; BMI, body mass index; DASH, Dietary Approaches to Stop Hypertension diet; HEI, Healthy Eating Index; n, sample size; NA, not applicable; NS, non-statistically significant (P-value>0.05); PBD, Plant-based diet; PMD, Panagiotakos Mediterranean diet; Q, quartile; S, statistically significant (P-value<0.05); TMD, Trichopoulou Mediterranean diet.

© 2023 Salas-Huetos A et al. *JAMA Network Open*.

eTable 4. Baseline Demographic and Reproductive Characteristics of Study Participants Who Underwent IVF and IUI Cycles, Overall, and in the Lowest and Highest Quartiles of Women's Adherence to the Different Dietary Patterns

	Overall	TMD		AMD		PMD		HEI		AHEI		AHA		DASH		PBD	
		Q1 (0-3)	Q4 (6-9)	Q1 (0-2)	Q4 (6-9)	Q1 (17-26)	Q4 (33-44)	Q1 (43.0-60.4)	Q4 (72.8-86.7)	Q1 (23-51)	Q4 (66-89)	Q1 (15-41)	Q4 (58-79)	Q1 (13-20)	Q4 (27-36)	Q1 (22-32)	Q4 (40-56)
n	612	194	188	118	178	147	151	147	160	145	159	154	153	159	171	176	134
Female demographic characteristics																	
Age (y), median (IQR)	35.0 (32.0, 38.0)	34.0 (31.0, 37.5)	35.0 (32.0, 38.0)	34.0 (31.0, 37.0)	35.0 (32.0, 38.0)[a]	35.0 (32.0, 38.0)	34.0 (32.0, 37.0)	34.0 (31.0, 37.0)	36.0 (33.0, 39.0)[a]	34.0 (31.0, 37.0)	35.0 (32.0, 38.0)[a]	34.0 (31.0, 37.0)	35.0 (32.0, 38.0)	34.0 (31.0, 38.0)	35.0 (32.0, 38.0)[a]	35.0 (32.0, 38.0)	34.5 (32.0, 38.0)
BMI (kg/m^2), median (IQR)	23.4 (21.3, 26.4)	23.4 (21.6, 26.3)	23.1 (21.2, 26.4)	23.7 (21.9, 26.3)	23.5 (21.2, 26.5)	23.9 (21.6, 26.3)	22.3 (20.8, 26.1)[a]	23.5 (21.2, 26.2)	22.7 (21.0, 25.9)	23.4 (21.4, 26.1)	23.2 (21.2, 25.9)	24.4 (22.1, 27.6)	22.7 (21.0, 25.7)[a]	23.9 (21.5, 26.4)	22.5 (20.7, 25.2)[a]	24.0 (21.6, 26.2)	22.7 (20.1, 26.4)
Race, white, n (%)	505 (82.8)	158 (81.9)	149 (79.7)	91 (77.8)	150 (84.8)	118 (80.3)	121 (80.7)	127 (86.4)	130 (81.8)	119 (82.1)	134 (84.8)	136 (88.9)	117 (77.0)[a]	130 (81.8)	141 (82.9)	141 (80.6)	106 (79.7)
Smoking status, ever smoker, n (%)	151 (24.7)	38 (19.7)	47 (25.0)[a]	26 (22.2)	44 (24.7)	41 (27.9)	34 (22.5)	35 (23.8)	33 (20.6)	39 (26.9)	40 (25.2)	42 (27.5)	37 (24.2)	42 (26.4)	43 (25.2)	50 (28.6)	31 (23.1)
Education, college or higher, n (%)	538 (92.1)	170 (93.4)	173 (94.0)	97 (89.0)	166 (94.7)	121 (89.0)	144 (98.0)[a]	119 (88.8)	146 (93.0)	118 (85.5)	148 (95.5)[a]	129 (89.6)	144 (96.0)	128 (87.7)	152 (92.1)	148 (92.5)	119 (92.3)
Moderate-to-vigorous physical activity (min/week)[b], median (IQR)	135.5 (29.5, 308.0)	108.8 (7.5, 300.0)	179.5 (89.0, 390.0)[a]	89.8 (2.5, 300.0)	180.0 (89.5, 360.0)[a]	77.0 (0.0, 240.0)	179.5 (72.0, 390.0)[a]	101.5 (0.0, 270.0)	179.5 (72.0, 390.0)[a]	90.0 (2.5, 240.0)	174.0 (83.0, 346.5)[a]	77.5 (2.5, 210.0)	174.0 (73.5, 359.0)[a]	90.0 (5.0, 222.0)	180.0 (78.5, 390.0)[a]	90.0 (1.3, 313.3)	156.0 (72.0, 360.0)[a]
Female energy intake (kcal/day), median (IQR)	1688 (1364, 2076)	1430 (1187, 1709)	1955 (1640, 2344)[a]	1420 (1100, 1728)	1956 (1667, 2328)[a]	1557 (1216, 1888)	1973 (1642, 2337)[a]	1631 (1356, 2046)	1756 (1465, 2204)[a]	1606 (1304, 1983)	1806 (1538, 2194)[a]	1535 (1212, 1889)	1856 (1615, 2219)[a]	1510 (1187, 1921)	1801 (1501, 2200)[a]	1499 (1214, 1795)	188.4 (1594, 2278)[a]
Multivitamin use (tablets/week), median (IQR)	3 (3, 3)	3 (3, 3)	3 (3, 3)	3 (2, 3)	3 (3, 3)	3 (2, 3)	3 (3, 3)	3 (2, 3)	3 (3, 3)	3 (2, 3)	3 (3, 3)	3 (3, 3)	3 (3, 3)	3 (2, 3)	3 (3, 3)	3 (2, 3)	3 (3, 3)
Couple cycle characteristics																	
Infertility diagnosis, n (%)		NA	NS	NA	NS	NA	NS	NA	NS	NA	NS	NA	NS	NA	NS	NA	NS
Female factor	235 (38.7)	71 (37.2)	80 (42.8)	45 (39.1)	75 (42.4)	62 (42.5)	70 (46.4)	55 (37.7)	63 (39.6)	58 (40.6)	64 (40.5)	56 (36.8)	63 (41.5)	60 (38.0)	66 (38.6)	72 (41.4)	60 (44.8)
Male factor	178 (29.1)	55 (28.8)	48 (25.7)	30 (26.1)	42 (23.7)	41 (28.1)	36 (23.8)	46 (31.5)	41 (25.8)	39 (27.3)	39 (24.7)	43 (28.3)	41 (27.0)	42 (26.6)	52 (30.4)	43 (24.7)	35 (26.1)
Unexplained	194 (32.0)	65 (34.0)	59 (31.6)	40 (34.8)	60 (33.9)	43 (29.5)	45 (29.8)	45 (30.8)	55 (34.6)	46 (32.2)	55 (34.8)	53 (34.9)	48 (31.6)	56 (35.4)	53 (31.0)	59 (33.9)	39 (29.1)
Treatment protocol, n (%)		NA	NS	NA	NS	NA	NS	NA	NS	NA	NS	NA	NS	NA	NS	NA	NS
Antagonist	48 (7.8)	12 (6.2)	15 (8.0)	5 (4.2)	12 (6.7)	12 (8.2)	12 (8.0)	10 (6.8)	16 (10.0)	9 (6.2)	13 (8.2)	9 (5.8)	10 (6.5)	10 (6.3)	14 (8.2)	16 (9.1)	12 (9.0)
Flare[c]	41 (6.7)	11 (5.7)	13 (6.9)	8 (6.8)	14 (7.9)	9 (6.1)	6 (4.0)	7 (4.8)	13 (8.1)	8 (5.5)	10 (6.3)	7 (4.6)	12 (7.8)	10 (6.3)	10 (5.9)	12 (6.8)	8 (6.0)
Luteal phase agonist[d]	484 (79.1)	153 (78.9)	150 (79.8)	96 (81.4)	142 (79.8)	114 (77.6)	125 (82.8)	122 (83.0)	121 (75.6)	118 (81.4)	127 (79.9)	128 (83.1)	121 (79.1)	130 (81.8)	135 (79.0)	134 (76.1)	105 (78.4)

© 2023 Salas-Huetos A et al. *JAMA Network Open*.

Egg donor or cryo cycle	39 (6.4)	18 (9.3)	10 (5.3)	9 (7.6)	10 (5.6)	12 (8.2)	8 (5.3)	8 (5.4)	10 (6.3)	10 (6.9)	9 (5.7)	10 (6.5)	10 (6.5)	9 (5.7)	12 (7.0)	14 (8.0)	9 (6.7)
Male demographic characteristics																	
Age (y), median (IQR)	36.3 (32.7, 39.8)	34.4 (31.7, 38.4)	36.6 (33.0, 40.6) [a]	34.2 (32.1)	35.9 (32.9, 40.5) [a]	35.4 (32.6, 38.7)	36.5 (33.2, 40.5)	34.4 (31.9, 38.5)	37.8 (34.4, 41.4) [a]	35.5 (32.1, 38.5)	35.6 (33.3, 39.8)	34.4 (32.1, 38.4)	36.4 (33.2, 39.9)	34.7 (31.7, 38.6)	36.7 (32.8, 39.5) [a]	35.5 (32.1, 39.4)	36.6 (33.0, 40.6)
BMI (kg/m^2), median (IQR)	27.1 (24.4, 29.8)	27.4 (24.8, 29.9)	26.3 (23.7, 29.0)	27.4 (24.8)	26.8 (24.4, 30.5)	28.1 (25.3, 30.7)	26.5 (24.2, 29.1)	27.8 (25.4, 30.7)	26.6 (23.8, 29.4)	28.0 (25.4, 30.0)	26.6 (24.0, 30.4)	27.8 (25.4, 30.0)	27.1 (24.7, 29.1)	27.5 (24.5, 30.6)	26.3 (24.3, 29.9)	27.6 (24.7, 29.8)	26.2 (24.3, 28.1)
Race, white, n (%)	307 (88.5)	107 (88.4)	90 (90.9)	74 (88.1)	86 (88.7)	66 (89.2)	96 (87.3)	84 (88.4)	74 (89.2)	89 (89.0)	84 (93.3)	87 (92.6)	75 (86.2)	79 (85.9)	81 (88.0)	92 (88.5)	59 (80.8)
Smoking status, ever smoker, n (%)	113 (32.5)	40 (33.1)	29 (29.0)	28 (33.3)	27 (27.6)	26 (35.1)	33 (29.7)	32 (33.7)	26 (31.0)	36 (36.0)	29 (31.9)	31 (33.0)	24 (27.3)	29 (31.5)	27 (29.0)	34 (32.7)	14 (18.9) [a]
Education, college or higher, n (%)	244 (85.6)	86 (83.5)	77 (92.8)	59 (83.1)	70 (92.1)	47 (81.0)	86 (92.5)	62 (81.6)	59 (88.1)	64 (78.1)	64 (88.9)	65 (85.5)	65 (94.2) [a]	59 (81.9)	67 (88.2)	75 (89.3)	51 (85.0)
Moderate-to-vigorous physical activity (min/week) [b], median (IQR)	149.5 (0.0, 369.8)	149.0 (12.0, 389.5)	169.0 (39.3, 390.0)	137.3 (0.0, 389.5)	158.3 (0.0, 390.0)	60.0 (0.0, 324.0)	161.5 (29.5, 390.0)	64.0 (0.0, 336.0)	138.0 (0.0, 369.8)	103.0 (0.0, 345.0)	150.0 (0.0, 359.0)	109.0 (0.0, 298.5)	126.0 (0.0, 329.3)	115.5 (0.0, 389.8)	119.0 (14.5, 332.5)	157.3 (0.0, 410.0)	149.8 (12.0, 383.0)

Data are presented as median (interquartile range) for continuous variables or n (%) for categorical variables. P-values were calculated using a Kruskal-Wallis test for continuous variables and a Chi-square test for categorical variables.

[a] P<0.05 for comparison of quartile 4 versus quartile 1 (reference). [b] Includes weight and aerobic exercise and sports. [c] Follicular-phase GnRH agonist/flare protocol. [d] Luteal-phase GnRH agonist protocol.

Abbreviations: AHA, American Heart Association diet recommendations; AHEI, Alternate Healthy Eating Index; AMD, Alternate Mediterranean diet; ART, assisted reproductive technologies; BMI, body mass index; DASH, Dietary Approaches to Stop Hypertension diet; HEI, Healthy Eating Index; IQR, interquartile range; n, sample size; NA, not applicable; NS, non-statistically significant (P-value>0.05); PBD, Plant-based diet; PMD, Panagiotakos Mediterranean diet; Q, quartile; S, statistically significant (P-value<0.05); TMD, Trichopoulou Mediterranean diet.

© 2023 Salas-Huetos A et al. *JAMA Network Open*.

eTable 5. Association Between Women's Adherence to A Priori–Defined Dietary Patterns According to Quartiles of Distribution and Probability of Clinical Pregnancy and Live Birth Following IVF or IUI Cycles[a]

		Number of women (n=612)/cycles (n=1572)	Clinical pregnancy, adjusted proportions (95% CI)	Live birth, adjusted proportions (95% CI)
**TMD**	**Q1**	194/496	0.35 (0.31, 0.40)	0.27 (0.23, 0.31)
	**Q2**	114/285	0.32 (0.26, 0.38)	0.24 (0.19, 0.30)
	**Q3**	116/294	0.34 (0.28, 0.40)	0.27 (0.22, 0.33)
	**Q4**	188/497	0.32 (0.27, 0.37)	0.26 (0.22, 0.30)
	**P trend**		0.38	0.81
**AMD**	**Q1**	118/308	0.35 (0.29, 0.41)	0.26 (0.21, 0.32)
	**Q2**	201/499	0.34 (0.29, 0.39)	0.26 (0.22, 0.30)
	**Q3**	115/299	0.34 (0.28, 0.40)	0.28 (0.23, 0.34)
	**Q4**	178/497	0.31 (0.27, 0.37)	0.25 (0.21, 0.30)
	**P trend**		0.43	0.80
**PMD**	**Q1**	147/421	0.31 (0.26, 0.36)	0.23 (0.19, 0.27)
	**Q2**	151/339	0.36 (0.30, 0.42)	0.29 (0.24, 0.34)
	**Q3**	163/403	0.35 (0.30, 0.40)	0.28 (0.23, 0.33)
	**Q4**	151/409	0.32 (0.27, 0.38)	0.25 (0.21, 0.30)
	**P trend**		0.76	0.45
**HEI**	**Q1**	147/393	0.35 (0.29, 0.40)	0.26 (0.21, 0.31)
	**Q2**	150/393	0.31 (0.26, 0.36)	0.25 (0.20, 0.30)
	**Q3**	155/393	0.33 (0.28, 0.39)	0.27 (0.22, 0.32)
	**Q4**	160/393	0.35 (0.30, 0.40)	0.27 (0.22, 0.32)
	**P trend**		0.90	0.62
**AHEI**	**Q1**	145/380	0.36 (0.31, 0.42)	0.27 (0.22, 0.32)
	**Q2**	145/383	0.33 (0.28, 0.39)	0.26 (0.21, 0.31)
	**Q3**	163/418	0.32 (0.27, 0.37)	0.25 (0.20, 0.29)
	**Q4**	159/391	0.33 (0.27, 0.38)	0.27 (0.22, 0.32)
	**P trend**		0.29	0.84
**AHA**	**Q1**	154/421	0.32 (0.27, 0.37)	0.22 (0.18, 0.27)
	**Q2**	139/346	0.37 (0.31, 0.43)	0.29 (0.24, 0.35) [b]
	**Q3**	166/404	0.33 (0.28, 0.39)	0.27 (0.23, 0.32)
	**Q4**	153/401	0.32 (0.27, 0.37)	0.26 (0.21, 0.31)
	**P trend**		0.80	0.39
[illegible]	**Q1**	159/421	0.34 (0.29, 0.39)	0.25 (0.21, 0.30)

© 2023 Salas-Huetos A et al. *JAMA Network Open*.

	Q2	122/324	0.32 (0.26, 0.38)	0.25 (0.20, 0.30)
	Q3	160/393	0.36 (0.30, 0.41)	0.28 (0.23, 0.33)
	Q4	171/434	0.32 (0.27, 0.37)	0.26 (0.22, 0.31)
	P trend		0.88	0.66
PBD	Q1	176/432	0.34 (0.29, 0.40)	0.26 (0.22, 0.31)
	Q2	115/323	0.33 (0.27, 0.39)	0.26 (0.21, 0.32)
	Q3	187/443	0.34 (0.29, 0.39)	0.28 (0.23, 0.32)
	Q4	134/374	0.32 (0.27, 0.38)	0.23 (0.19, 0.28)
	P trend		0.63	0.45

Data is presented as predicted marginal proportions and 95% confidence intervals. Analyses were run using generalized linear mixed models (proc glimmix) with random intercepts, binary distribution, and logit link.

[a] Fully adjusted model: Female age, BMI, physical activity, and energy intake. [b] P<0.05 for comparison of specific quartile versus quartile 1 (reference).

Abbreviations: AHA, American Heart Association Index; AHEI, Alternate Healthy Eating Index; AMD, Alternate Mediterranean diet; ART, assisted reproductive technologies; CI, confidence interval; DASH, Dietary Approach to Stop Hypertension index; IUI, Intra-uterine insemination; HEI, Healthy Eating Index; n, sample size; PBD, Plant-based diet; PMD, Panagiotakos Mediterranean diet; Q, quartile; TMD, Trichopoulou Mediterranean diet.

© 2023 Salas-Huetos A et al. *JAMA Network Open*.

eTable 6. Sensitivity Analysis: Association Between Women's Adherence to A Priori–Defined Dietary Patterns According to Quartiles of Distribution and Probability of Clinical Pregnancy and Live Birth Following IVF After Exclusion of Canceled Cycles[a]

		Number of women (n=344)/cycles (n=511)	Clinical pregnancy, adjusted proportions (95% CI)	Live birth, adjusted proportions (95% CI)
**TMD**	**Q1**	106/159	0.50 (0.41, 0.59)	0.41 (0.33, 0.50)
	**Q2**	58/89	0.57 (0.45, 0.68)	0.50 (0.38, 0.61)
	**Q3**	66/102	0.59 (0.48, 0.69)	0.44 (0.39, 0.57)
	**Q4**	114/161	0.58 (0.49, 0.67)	0.48 (0.39, 0.57)
	**P trend**		0.18	0.32
**AMD**	**Q1**	65/95	0.54 (0.42, 0.65)	0.45 (0.34, 0.56)
	**Q2**	102/151	0.56 (0.47, 0.64)	0.44 (0.36, 0.53)
	**Q3**	75/115	0.51 (0.41, 0.61)	0.44 (0.34, 0.54)
	**Q4**	102/150	0.60 (0.51, 0.69)	0.48 (0.39, 0.58)
	**P trend**		0.40	0.60
**PMD**	**Q1**	77/126	0.50 (0.40, 0.59)	0.40 (0.31, 0.50)
	**Q2**	79/113	0.63 (0.53, 0.72)	0.53 (0.43, 0.62)
	**Q3**	99/145	0.58 (0.49, 0.67)	0.46 (0.37, 0.55)
	**Q4**	89/127	0.53 (0.43, 0.62)	0.43 (0.34, 0.53)
	**P trend**		0.77	0.87
**HEI**	**Q1**	85/134	0.54 (0.44, 0.63)	0.42 (0.33, 0.51)
	**Q2**	82/116	0.58 (0.48, 0.68)	0.48 (0.38, 0.58)
	**Q3**	84/123	0.54 (0.44, 0.63)	0.45 (0.36, 0.55)
	**Q4**	93/138	0.57 (0.48, 0.66)	0.47 (0.38, 0.57)
	**P trend**		0.73	0.50
**AHEI**	**Q1**	87/135	0.56 (0.47, 0.65)	0.45 (0.36, 0.55)
	**Q2**	93/134	0.57 (0.48, 0.66)	0.48 (0.39, 0.58)
	**Q3**	72/112	0.55 (0.44, 0.65)	0.42 (0.33, 0.52)
	**Q4**	92/130	0.54 (0.45, 0.64)	0.45 (0.36, 0.55)
	**P trend**		0.71	0.78
**AHA**	**Q1**	84/140	0.49 (0.40, 0.59)	0.38 (0.30, 0.48)
	**Q2**	77/109	0.64 (0.53, 0.73) [b]	0.51 (0.41, 0.62)
	**Q3**	98/137	0.59 (0.50, 0.68)	0.48 (0.39, 0.58)
	**Q4**	85/125	0.52 (0.42, 0.62)	0.44 (0.35, 0.54)
	**P trend**		0.79	0.42
**DASH**	**Q1**	82/129	0.56 (0.46, 0.65)	0.46 (0.37, 0.56)

© 2023 Salas-Huetos A et al. *JAMA Network Open*.

	**Q2**	66/97	0.54 (0.43, 0.65)	0.43 (0.33, 0.54)
	**Q3**	97/146	0.57 (0.48, 0.65)	0.44 (0.36, 0.53)
	**Q4**	99/139	0.56 (0.47, 0.65)	0.47 (0.38, 0.57)
	**P trend**		0.92	0.82
**PBD**	**Q1**	74/117	0.49 (0.39, 0.59)	0.39 (0.30, 0.49)
	**Q2**	86/127	0.55 (0.45, 0.64)	0.49 (0.39, 0.58)
	**Q3**	112/160	0.56 (0.48, 0.65)	0.46 (0.38, 0.55)
	**Q4**	72/107	0.63 (0.53, 0.73)	0.47 (0.37, 0.58)
	**P trend**		0.07	0.39

Data is presented as predicted marginal proportions and 95% confidence intervals. Analyses were run using generalized linear mixed models (proc glimmix) with random intercepts, binary distribution, and logit link.

[a] Fully adjusted model: Female age, BMI, physical activity, energy intake, day of transference, and number of embryos transferred. [b] P<0.05 for comparison of specific quartile versus quartile 1 (reference).

Abbreviations: AHA, American Heart Association Index; AHEI, Alternate Healthy Eating Index; AMD, Alternate Mediterranean diet; CI, confidence interval; DASH, Dietary Approach to Stop Hypertension index; HEI, Healthy Eating Index; IVF, *in vitro* fertilization; n, sample size; PBD, Plant-based diet; PMD, Panagiotakos Mediterranean diet; Q, quartile; TMD, Trichopoulou Mediterranean diet.

© 2023 Salas-Huetos A et al. *JAMA Network Open*.

eTable 7. Sensitivity Analysis: Association Between Women's Adherence to A Priori–Defined Dietary Patterns According to Quartiles of Distribution and Probability of Implantation, Clinical Pregnancy, and Live Birth Following Infertility Treatment (IUI or IVF Cycles) After Exclusion of Egg Donor Cycles[a]

		Number of women (n=297)/cycles (n=739)	Clinical pregnancy, adjusted proportions (95% CI)	Live birth, adjusted proportions (95% CI)
**TMD**	**Q1**	99/238	0.48 (0.41, 0.55)	0.38 (0.32, 0.46)
	**Q2**	58/142	0.48 (0.39, 0.56)	0.38 (0.30, 0.47)
	**Q3**	49/136	0.52 (0.43, 0.60)	0.41 (0.33, 0.51)
	**Q4**	91/223	0.50 (0.43, 0.57)	0.42 (0.35, 0.49)
	**P trend**		0.64	0.49
**AMD**	**Q1**	60/148	0.47 (0.39, 0.56)	0.39 (0.31, 0.48)
	**Q2**	94/228	0.51 (0.44, 0.58)	0.40 (0.33, 0.47)
	**Q3**	61/164	0.45 (0.38, 0.53)	0.39 (0.32, 0.47)
	**Q4**	82/199	0.51 (0.44, 0.58)	0.42 (0.34, 0.49)
	**P trend**		0.63	0.70
**PMD**	**Q1**	69/191	0.46 (0.39, 0.54)	0.36 (0.29, 0.44)
	**Q2**	71/161	0.50 (0.42, 0.58)	0.43 (0.35, 0.52)
	**Q3**	86/213	0.51 (0.44, 0.58)	0.41 (0.34, 0.48)
	**Q4**	71/174	0.49 (0.41, 0.57)	0.41 (0.33, 0.49)
	**P trend**		0.58	0.50
**HEI**	**Q1**	70/171	0.50 (0.42, 0.58)	0.40 (0.32, 0.48)
	**Q2**	76/174	0.49 (0.41, 0.57)	0.41 (0.33, 0.49)
	**Q3**	77/207	0.46 (0.39, 0.53)	0.38 (0.31, 0.45)
	**Q4**	74/187	0.51 (0.44, 0.59)	0.42 (0.35, 0.50)
	**P trend**		0.96	0.76
**AHEI**	**Q1**	70/164	0.54 (0.47, 0.62)	0.44 (0.36, 0.52)
	**Q2**	72/179	0.50 (0.42, 0.58)	0.40 (0.33, 0.48)
	**Q3**	78/213	0.45 (0.38, 0.52)	0.37 (0.30, 0.44)
	**Q4**	77/183	0.48 (0.40, 0.55)	0.40 (0.33, 0.48)
	**P trend**		0.16	0.42
**AHA**	**Q1**	72/197	0.44 (0.37, 0.52)	0.34 (0.27, 0.41)
	**Q2**	72/169	0.54 (0.46, 0.62)	0.44 (0.36, 0.52)
	**Q3**	86/198	0.52 (0.44, 0.59)	0.43 (0.36, 0.50)
	**Q4**	67/175	0.47 (0.39, 0.54)	0.40 (0.32, 0.48)
	**P trend**		0.75	0.30
**[illegible]**	**Q1**	73/184	0.51 (0.44, 0.59)	0.42 (0.35, 0.50)

© 2023 Salas-Huetos A et al. *JAMA Network Open*.

	**Q2**	59/154	0.46 (0.38, 0.55)	0.37 (0.29, 0.46)
	**Q3**	86/211	0.50 (0.43, 0.57)	0.40 (0.33, 0.47)
	**Q4**	79/190	0.48 (0.41, 0.56)	0.41 (0.33, 0.49)
	**P trend**		0.69	0.86
**PBD**	**Q1**	87/207	0.47 (0.40, 0.54)	0.40 (0.33, 0.47)
	**Q2**	53/190	0.48 (0.39, 0.56)	0.40 (0.32, 0.49)
	**Q3**	91/175	0.50 (0.43, 0.57)	0.43 (0.36, 0.50)
	**Q4**	66/167	0.51 (0.43, 0.59)	0.37 (0.30, 0.46)
	**P trend**		0.42	0.84

Data is presented as predicted marginal proportions and 95% confidence intervals. Analyses were run using generalized linear mixed models (proc glimmix) with random intercepts, binary distribution, and logit link.

[a] Fully adjusted model: Female age, BMI, physical activity, and energy intake. [b] P<0.05 for comparison of specific quartile versus quartile 1 (reference).

Abbreviations: AHA, American Heart Association Index; AHEI, Alternate Healthy Eating Index; AMD, Alternate Mediterranean diet; ART, assisted reproductive technologies; CI, confidence interval; DASH, Dietary Approach to Stop Hypertension index; IUI, Intra-uterine insemination; HEI, Healthy Eating Index; n, sample size; PBD, Plant-based diet; PMD, Panagiotakos Mediterranean diet; Q, quartile; TMD, Trichopoulou Mediterranean diet.

© 2023 Salas-Huetos A et al. *JAMA Network Open*.

eTable 8. Association Between Women's Adherence to the Different Dietary Patterns According to Quartiles of Distribution and the Adjusted Means of Early Infertility Treatment Outcomes: Estradiol Trigger Levels and Endometrial Thickness[a]

		Estradiol trigger levels (pmol/L) (number of cycles=571[b]), adjusted means (95% CI)	Endometrial thickness (mm) (number of cycles=571[b]), adjusted means (95% CI)
TMD	**Q1**	2285 (2123, 2448)	10.6 (10.2, 11.0)
	**Q2**	2159 (1954, 2364)	10.3 (9.8, 10.8)
	**Q3**	2051 (1849, 2252)	10.2 (9.7, 10.8)
	**Q4**	2117 (1956, 2278)	10.2 (9.8, 10.6)
	**P trend**	0.10	0.19
AMD	**Q1**	2230 (2023, 2436)	10.3 (9.8, 10.9)
	**Q2**	2155 (1994, 2316)	10.4 (9.9, 10.8)
	**Q3**	2182 (1993, 2370)	10.5 (10.0, 11.0)
	**Q4**	2119 (1949, 2289)	10.2 (9.8, 10.7)
	**P trend**	0.46	0.80
PMD	**Q1**	2414 (2234, 2594)	10.5 (10.0, 10.9)
	**Q2**	2013 (1834, 2193)[c]	10.3 (9.8, 10.7)
	**Q3**	2145 (1984, 2306)[c]	10.3 (9.9, 10.7)
	**Q4**	2088 (1909, 2267)[c]	10.4 (9.9, 10.9)
	**P trend**	**0.04**	0.83
HEI	**Q1**	2176 (1994, 2358)	10.3 (9.8, 10.7)
	**Q2**	2001 (1813, 2190)	10.4 (9.9, 10.9)
	**Q3**	2274 (2107, 2443)	10.5 (10.1, 10.9)
	**Q4**	2174 (2007, 2340)	10.3 (9.8, 10.7)
	**P trend**	0.62	0.89
AHEI	**Q1**	2169 (1988, 2351)	10.2 (9.7, 10.7)
	**Q2**	2242 (2064, 2419)	10.4 (10.0, 10.9)
	**Q3**	2150 (1980, 2320)	10.5 (10.1, 11.0)
	**Q4**	2101 (1927, 2274)	10.2 (9.8, 10.7)
	**P trend**	0.46	0.83
AHA	**Q1**	2111 (1935, 2286)	10.1 (9.7, 10.6)
	**Q2**	2197 (2013, 2381)	10.3 (9.8, 10.8)
	**Q3**	2175 (2007, 2343)	11.0 (10.5, 11.4)[c]
	**Q4**	2178 (1998, 2359)	9.9 (9.5, 10.4)
	**P trend**	0.64	0.99
[illegible]	**Q1**	2149 (1968, 2329)	10.2 (9.7, 10.7)

© 2023 Salas-Huetos A et al. *JAMA Network Open*.

	**Q2**	2250 (2047, 2452)	10.7 (10.2, 11.3)
	**Q3**	2142 (1977, 2307)	10.4 (10.0, 10.8)
	**Q4**	2143 (1975, 2311)	10.1 (9.7, 10.6)
	**P trend**	0.82	0.63
**PBD**	**Q1**	2143 (1973, 2313)	10.3 (9.8, 10.7)
	**Q2**	2148 (1949, 2348)	10.3 (9.8, 10.9)
	**Q3**	2175 (2017, 2332)	10.5 (10.1, 10.9)
	**Q4**	2190 (2002, 2379)	10.2 (9.7, 10.7)
	**P trend**	0.69	0.96

Data is presented as adjusted means and 95% confidence intervals. Analyses were run using generalized linear mixed models (proc mixed) with random intercepts, normal distribution and identity link function.

[a] Fully adjusted model: Female age, BMI, physical activity, and energy intake. [b] Fresh ART cycles. [c] $P<0.05$ for comparison of specific quartile versus quartile 1 (reference).

Abbreviations: AHA, American Heart Association Index; AHEI, Alternate Healthy Eating Index; AMD, Alternate Mediterranean diet; ART, assisted reproductive technologies; CI, confidence interval; DASH, Dietary Approach to Stop Hypertension index; IVF, in vitro fertilization; HEI, Healthy Eating Index; n, sample size; PBD, Plant-based diet; PMD, Panagiotakos Mediterranean diet; Q, quartile; TMD, Trichopoulou Mediterranean diet.

© 2023 Salas-Huetos A et al. *JAMA Network Open*.

eTable 9. Baseline Women's Food Groups Information in the Lowest and Highest Quartiles of Women's Adherence to the Different Dietary Patterns Following Infertility Treatment With IUI or IVF

	Overall	TMD		AMD		PMD		HEI		AHEI		AHA		DASH		PBD	
		Q1 (0-3)	Q4 (6-9)	Q1 (0-2)	Q4 (6-9)	Q1 (17-26)	Q4 (33-43)	Q1 (43.8-60.6)	Q4 (72.8-86.1)	Q1 (23-52)	Q4 (66-89)	Q1 (15-41)	Q4 (58-79)	Q1 (13-20)	Q4 (27-36)	Q1 (22-31)	Q4 (40-51)
n	612	194	188	118	178	147	151	147	160	145	159	154	153	159	171	176	134
Fruit (servings/day), median (IQR)	1.23 (0.70, 1.83)	0.81 (0.52, 1.32)	1.79 (1.23, 2.32)[a]	0.68 (0.46, 1.00)	1.85 (1.38, 2.43)[a]	0.67 (0.42, 1.20)	1.85 (1.26, 2.45)[a]	0.62 (0.36, 1.29)	1.79 (1.27, 2.31)[a]	0.77 (0.44, 1.25)	1.73 (1.23, 2.42)[a]	0.68 (0.44, 1.07)	1.91 (1.40, 2.51)[a]	0.62 (0.42, 1.01)	1.87 (1.37, 2.45)[a]	0.77 (0.56, 1.30)	1.64 (1.19, 2.45)[a]
Fruit juice (servings/day), median (IQR)	0.16 (0.06, 0.49)	0.16 (0.06, 0.49)	0.18 (0.06, 0.53)	0.14 (0.06, 0.32)	0.16 (0.06, 0.49)	0.16 (0.06, 0.38)	0.20 (0.06, 0.57)	0.22 (0.08, 0.49)	0.15 (0.05, 0.49)	0.32 (0.14, 0.90)	0.08 (0.02, 0.18)[a]	0.18 (0.08, 0.49)	0.16 (0.04, 0.49)	0.18 (0.08, 0.40)	0.16 (0.06, 0.52)	0.14 (0.06, 0.30)	0.17 (0.06, 0.57)[a]
Crucif (servings/day), median (IQR)	0.28 (0.16, 0.52)	0.18 (0.10, 0.32)	0.40 (0.26, 0.78)[a]	0.16 (0.10, 0.28)	0.46 (0.26, 0.82)[a]	0.16 (0.10, 0.28)	0.46 (0.24, 0.81)[a]	0.16 (0.10, 0.32)	0.40 (0.22, 0.67)[a]	0.16 (0.10, 0.28)	0.46 (0.28, 0.86)[a]	0.16 (0.10, 0.28)	0.49 (0.26, 0.87)[a]	0.20 (0.10, 0.32)	0.40 (0.22, 0.81)[a]	0.22 (0.14, 0.40)	0.47 (0.24, 0.87)[a]
Yellow (servings/day), median (IQR)	0.30 (0.14, 0.59)	0.18 (0.10, 0.32)	0.59 (0.30, 0.90)[a]	0.17 (0.10, 0.26)	0.61 (0.32, 0.90)[a]	0.16 (0.10, 0.28)	0.59 (0.26, 0.94)[a]	0.18 (0.10, 0.38)	0.45 (0.19, 0.85)[a]	0.20 (0.10, 0.32)	0.55 (0.26, 0.86)[a]	0.18 (0.12, 0.32)	0.59 (0.26, 0.94)[a]	0.18 (0.10, 0.30)	0.55 (0.26, 0.90)[a]	0.20 (0.12, 0.32)	0.61 (0.32, 0.96)
Tomato (servings/day), median (IQR)	0.57 (0.30, 0.84)	0.34 (0.18, 0.59)	0.71 (0.45, 0.97)[a]	0.30 (0.18, 0.57)	0.67 (0.42, 1.00)[a]	0.36 (0.18, 0.61)	0.79 (0.55, 1.02)[a]	0.47 (0.24, 0.65)	0.65 (0.37, 0.96)[a]	0.38 (0.22, 0.65)	0.65 (0.42, 0.96)[a]	0.47 (0.24, 0.65)	0.71 (0.47, 0.98)[a]	0.44 (0.22, 0.61)	0.71 (0.45, 1.02)[a]	0.47 (0.24, 0.61)	0.71 (0.47, 1.00)[a]
Green (servings/day), median (IQR)	0.53 (0.26, 0.96)	0.32 (0.20, 0.59)	0.73 (0.38, 1.22)[a]	0.31 (0.18, 0.50)	0.73 (0.38, 1.16)[a]	0.36 (0.20, 0.67)	0.73 (0.44, 1.22)[a]	0.36 (0.20, 0.67)	0.72 (0.37, 1.14)[a]	0.32 (0.20, 0.67)	0.73 (0.36, 1.12)[a]	0.36 (0.24, 0.67)	0.71 (0.38, 1.22)[a]	0.32 (0.20, 0.67)	0.73 (0.44, 1.16)[a]	0.38 (0.20, 0.67)	0.79 (0.36, 1.18)[a]
Legume (servings/day), median (IQR)	0.24 (0.14, 0.50)	0.14 (0.08, 0.20)	0.52 (0.26, 0.79)[a]	0.12 (0.08, 0.20)	0.53 (0.26, 0.79)[a]	0.14 (0.08, 0.24)	0.61 (0.32, 0.96)[a]	0.12 (0.06, 0.20)	0.44 (0.24, 0.76)[a]	0.16 (0.10, 0.24)	0.44 (0.20, 0.73)[a]	0.16 (0.10, 0.24)	0.50 (0.26, 0.79)[a]	0.18 (0.10, 0.24)	0.47 (0.24, 0.88)[a]	0.18 (0.10, 0.26)	0.55 (0.30, 0.92)[a]
Potato (servings/day), median (IQR)	0.08 (0.02, 0.14)	0.08 (0.02, 0.14)	0.08 (0.02, 0.14)	0.08 (0.02, 0.08)	0.08 (0.02, 0.14)	0.08 (0.08, 0.14)	0.08 (0.02, 0.14)	0.08 (0.08, 0.14)	0.08 (0.02, 0.08)[a]	0.08 (0.08, 0.14)	0.08 (0.02, 0.08)[a]	0.08 (0.08, 0.14)	0.08 (0.02, 0.08)[a]	0.08 (0.08, 0.14)	0.08 (0.02, 0.08)[a]	0.08 (0.02, 0.08)	0.08 (0.08, 0.14)[a]
Grains (servings/day), median (IQR)	2.38 (1.62, 3.45)	1.93 (1.35, 2.57)	3.09 (2.32, 4.09)[a]	2.01 (1.32, 2.87)	2.99 (2.15, 4.20)[a]	2.01 (1.38, 3.06)	3.03 (2.21, 4.37)[a]	2.34 (1.50, 3.18)	2.72 (1.90, 3.95)[a]	2.40 (1.62, 3.34)	2.42 (1.61, 3.51)	2.16 (1.40, 3.06)	2.74 (1.91, 4.12)[a]	2.03 (1.36, 3.04)	2.63 (1.93, 3.93)[a]	1.81 (1.16, 2.48)	3.07 (2.30, 4.22)[a]
Whole grain (servings/day), median (IQR)	0.95 (0.47, 1.49)	0.63 (0.31, 1.04)	1.35 (0.75, 2.23)[a]	0.50 (0.28, 0.98)	1.44 (1.04, 2.31)[a]	0.59 (0.26, 1.02)	1.47 (0.88, 2.77)[a]	0.47 (0.24, 1.08)	1.45 (0.80, 2.59)[a]	0.63 (0.28, 1.14)	1.29 (0.67, 2.17)[a]	0.55 (0.27, 0.98)	1.45 (0.89, 2.59)[a]	0.47 (0.26, 1.04)	1.44 (0.91, 2.52)[a]	0.63 (0.30, 1.16)	1.24 (0.67, 2.19)[a]
Refined grain (servings/day), median (IQR)	0.84 (0.52, 1.30)	0.77 (0.47, 1.14)	1.00 (0.61, 1.61)[a]	0.84 (0.56, 1.26)	0.94 (0.58, 1.44)	0.81 (0.51, 1.26)	1.00 (0.64, 1.57)[a]	1.00 (0.58, 1.43)	0.74 (0.48, 1.19)[a]	1.03 (0.64, 1.36)	0.70 (0.46, 1.18)[a]	0.93 (0.56, 1.32)	0.81 (0.51, 1.34)	0.91 (0.58, 1.37)	0.79 (0.50, 1.28)	0.70 (0.45, 1.05)	1.10 (0.74, 1.71)[a]
Nuts (servings/day), median (IQR)	0.48 (0.18, 0.96)	0.24 (0.12, 0.51)	0.73 (0.32, 1.23)[a]	0.18 (0.10, 0.30)	0.89 (0.53, 1.24)[a]	0.30 (0.14, 0.71)	0.67 (0.30, 1.18)[a]	0.22 (0.10, 0.51)	0.90 (0.49, 1.39)[a]	0.20 (0.08, 0.32)	0.98 (0.61, 1.36)[a]	0.24 (0.12, 0.55)	0.73 (0.26, 1.22)[a]	0.20 (0.10, 0.47)	0.88 (0.44, 1.29)[a]	0.20 (0.08, 0.49)	0.73 (0.32, 1.29)[a]
Fish (servings/day), median (IQR)	0.21 (0.13, 0.33)	0.15 (0.08, 0.21)	0.28 (0.77, 1.68)[a]	0.15 (0.08, 0.20)	0.28 (0.20, 0.40)[a]	0.14 (0.04, 0.23)	0.29 (0.18, 0.41)[a]	0.18 (0.09, 0.27)	0.27 (0.15, 0.39)[a]	0.13 (0.06, 0.25)	0.28 (0.20, 0.40)[a]	0.13 (0.06, 0.20)	0.32 (0.20, 0.41)[a]	0.19 (0.11, 0.30)	0.24 (0.11, 0.38)	0.26 (0.15, 0.37)	0.16 (0.06, 0.28)[a]
Meat (servings/day), median (IQR)	1.13 (0.72, 1.59)	1.08 (0.77, 1.47)	1.26 (0.77, 1.68)	1.04 (0.78, 1.50)	1.25 (0.73, 1.68)	1.21 (0.85, 1.71)	1.09 (0.59, 1.63)	1.18 (0.80, 1.57)	1.03 (0.56, 1.55)	1.18 (0.81, 1.61)	1.06 (0.66, 1.55)	1.15 (0.80, 1.65)	1.12 (0.68, 1.59)	1.25 (0.90, 1.73)	0.93 (0.52, 1.48)[a]	1.30 (0.97, 1.73)	0.80 (0.46, 1.49)[a]
Processed meat (servings/day), median (IQR)	0.44 (0.21, 0.75)	0.50 (0.29, 0.81)	0.36 (0.16, 0.72)[a]	0.60 (0.33, 0.86)	0.34 (0.14, 0.64)[a]	0.55 (0.31, 0.89)	0.27 (0.05, 0.62)[a]	0.56 (0.31, 0.88)	0.27 (0.10, 0.61)[a]	0.62 (0.34, 0.89)	0.29 (0.09, 0.57)[a]	0.67 (0.40, 1.00)	0.26 (0.11, 0.44)[a]	0.62 (0.41, 0.94)	0.25 (0.06, 0.45)[a]	0.53 (0.31, 0.82)	0.29 (0.05, 0.61)[a]
Red meat (servings/day), median (IQR)	0.28 (0.10, 0.40)	0.28 (0.18, 0.40)	0.22 (0.06, 0.40)[a]	0.33 (0.22, 0.46)	0.22 (0.06, 0.38)[a]	0.28 (0.16, 0.52)	0.22 (0.02, 0.34)[a]	0.34 (0.22, 0.46)	0.16 (0.04, 0.33)[a]	0.34 (0.22, 0.52)	0.18 (0.04, 0.34)[a]	0.34 (0.22, 0.52)	0.16 (0.04, 0.34)[a]	0.34 (0.24, 0.58)	0.14 (0.02, 0.28)[a]	0.28 (0.19, 0.46)	0.18 (0.00, 0.34)[a]

© 2023 Salas-Huetos A et al. *JAMA Network Open*.

Chicken (servings/day), median (IQR)	0.42 (0.18, 0.59)	0.36 (0.18, 0.57)	0.44 (0.18, 0.59)	0.29 (0.18, 0.53)	0.45 (0.18, 0.65)	0.47 (0.24, 0.59)	0.36 (0.14, 0.59) [a]	0.36 (0.18, 0.57)	0.30 (0.16, 0.59)	0.36 (0.18, 0.59)	0.43 (0.16, 0.59)	0.30 (0.18, 0.57)	0.43 (0.16, 0.59)	0.45 (0.18, 0.59)	0.36 (0.14, 0.59)	0.45 (0.22, 0.59)	0.24 (0.10, 0.51) [a]
Low-fat dairy (servings/day), median (IQR)	0.67 (0.26, 1.08)	0.86 (0.28, 1.08)	0.63 (0.28, 1.12)	0.59 (0.20, 1.04)	0.77 (0.32, 1.16)	0.59 (0.20, 1.04)	0.63 (0.20, 1.18) [a]	0.55 (0.18, 1.00)	0.86 (0.40, 1.18) [a]	0.86 (0.43, 1.12)	0.61 (0.30, 1.04)	0.61 (0.20, 1.04)	0.73 (0.30, 1.12)	0.26 (0.12, 0.73)	0.96 (0.56, 1.45) [a]	0.59 (0.26, 1.02)	0.63 (0.22, 1.32) [a]
High-fat dairy (servings/day), median (IQR)	0.98 (0.55, 1.51)	1.05 (0.67, 1.92)	0.71 (0.46, 1.27) [a]	0.98 (0.63, 1.32)	0.88 (0.49, 1.32) [a]	1.16 (0.63, 2.26)	0.94 (0.43, 1.34) [a]	1.18 (0.69, 2.06)	0.66 (0.44, 1.20) [a]	1.08 (0.71, 1.67)	0.86 (0.49, 1.34) [a]	1.09 (0.69, 1.96)	0.71 (0.36, 1.20) [a]	1.04 (0.59, 1.61)	0.86 (0.49, 1.34) [a]	1.13 (0.67, 1.99)	0.74 (0.47, 1.32) [a]
Eggs (servings/day), median (IQR)	0.43 (0.16, 0.52)	0.28 (0.14, 0.45)	0.45 (0.16, 0.80) [a]	0.26 (0.10, 0.45)	0.43 (0.18, 0.82) [a]	0.24 (0.10, 0.45)	0.45 (0.18, 0.71) [a]	0.42 (0.14, 0.45)	0.45 (0.16, 0.80) [a]	0.22 (0.10, 0.43)	0.45 (0.16, 0.84) [a]	0.29 (0.14, 0.45)	0.45 (0.16, 0.80) [a]	0.43 (0.14, 0.45)	0.43 (0.16, 0.65) [a]	0.43 (0.16, 0.81)	0.22 (0.10, 0.45) [a]
High energy drinks (servings/day), median (IQR)	0.04 (0.02, 0.14)	0.04 (0.02, 0.14)	0.04 (0.02, 0.10)	0.08 (0.02, 0.16)	0.04 (0.02, 0.10) [a]	0.08 (0.02, 0.18)	0.04 (0.00, 0.10) [a]	0.10 (0.02, 0.22)	0.02 (0.01, 0.08) [a]	0.10 (0.04, 0.43)	0.02 (0.00, 0.04) [a]	0.10 (0.04, 0.24)	0.02 (0.00, 0.04) [a]	0.10 (0.04, 0.22)	0.02 (0.00, 0.08) [a]	0.04 (0.02, 0.10)	0.04 (0.00, 0.10)
Low energy drinks (servings/day), median (IQR)	0.04 (0.00, 0.17)	0.04 (0.00, 0.18)	0.04 (0.00, 0.14) [a]	0.08 (0.02, 0.24)	0.04 (0.00, 0.14) [a]	0.08 (0.02, 0.47)	0.02 (0.00, 0.12) [a]	0.08 (0.02, 0.51)	0.02 (0.00, 0.10) [a]	0.10 (0.02, 0.53	0.02 (0.00, 0.08) [a]	0.09 (0.02, 0.51)	0.02 (0.00, 0.08) [a]	0.08 (0.02, 0.51)	0.02 (0.00, 0.10) [a]	0.08 (0.02, 0.18)	0.04 (0.00, 0.14)
Sweets (servings/day), median (IQR)	0.60 (0.38, 1.07)	0.56 (0.36, 1.00)	0.61 (0.38, 1.09)	0.66 (0.40, 1.04)	0.60 (0.38, 1.11)	0.61 (0.32, 1.17)	0.52 (0.36, 0.99)	0.79 (0.46, 1.23)	0.45 (0.31, 0.85) [a]	0.67 (0.44, 1.12)	0.50 (0.30, 0.92) [a]	0.65 (0.38, 1.09)	0.50 (0.32, 0.93)	0.66 (0.40, 1.03)	0.52 (0.32, 0.97)	0.56 (0.30, 0.94)	0.60 (0.40, 1.20) [a]
Beer (servings/day), median (IQR)	0.08 (0.00, 0.17)	0.06 (0.00, 0.16)	0.08 (0.03, 0.17) [a]	0.06 (0.00, 0.14)	0.08 (0.03, 0.17) [a]	0.03 (0.00, 0.16)	0.08 (0.03, 0.16) [a]	0.08 (0.00, 0.17)	0.07 (0.00, 0.16)	0.03 (0.00, 0.16)	0.08 (0.00, 0.17) [a]	0.06 (0.00, 0.16)	0.06 (0.00, 0.16)	0.08 (0.02, 0.17)	0.06 (0.00, 0.16)	0.08 (0.00, 0.20)	0.06 (0.40, 1.20) [a]
Wine (servings/day), median (IQR)	0.16 (0.06, 0.51)	0.11 (0.03, 0.22)	0.28 (0.11, 0.57) [a]	0.08 (0.03, 0.17)	0.25 (0.11, 0.57) [a]	0.08 (0.00, 0.46)	0.28 (0.11, 0.57) [a]	0.11 (0.03, 0.46)	0.17 (0.06, 0.57) [a]	0.10 (0.00, 0.22)	0.28 (0.11, 0.86) [a]	0.11 (0.03, 0.46)	0.22 (0.08, 0.57) [a]	0.16 (0.06, 0.51)	0.16 (0.06, 0.51)	0.17 (0.06, 0.57)	0.16 (0.06, 0.51)
Liquor (servings/day), median (IQR)	0.03 (0.00, 0.08)	0.03 (0.00, 0.08)	0.03 (0.00, 0.08)	0.03 (0.00, 0.08)	0.03 (0.00, 0.08) [a]	0.03 (0.00, 0.08)	0.03 (0.00, 0.08)	0.03 (0.00, 0.08)	0.03 (0.00, 0.08)	0.03 (0.00, 0.03)	0.03 (0.00, 0.08) [a]	0.03 (0.00, 0.08)	0.03 (0.00, 0.08)	0.03 (0.00, 0.08)	0.03 (0.00, 0.08)	0.03 (0.00, 0.08)	0.03 (0.00, 0.08)

Data are presented as median (interquartile range). P-values were calculated using a Kruskal-Wallis test.

[a] P<0.05 for comparison of quartile 4 versus quartile 1 (reference).

Abbreviations: AHA, American Heart Association diet recommendations; AHEI, Alternate Healthy Eating Index; AMD, Alternate Mediterranean diet; DASH, Dietary Approaches to Stop Hypertension diet; HEI, Healthy Eating Index; IQR, interquartile range; n, sample size; PBD, Plant-based diet; PMD, Panagiotakos Mediterranean diet; Q, quartile; TMD, Trichopoulou Mediterranean diet.

© 2023 Salas-Huetos A et al. *JAMA Network Open*.

eTable 10. Baseline Women's Reproductive Essential Micronutrients Intake Information in the Lowest and Highest Quartiles of Women's Adherence to the Different Dietary Patterns Following Infertility Treatment With IUI or IVF

	Overall	TMD		AMD		PMD		HEI		AHEI		AHA		DASH		PBD	
		Q1 (0-3)	Q4 (6-9)	Q1 (0-2)	Q4 (6-9)	Q1 (17-26)	Q4 (33-43)	Q1 (43.8-60.6)	Q4 (72.8-86.1)	Q1 (23-52)	Q4 (66-89)	Q1 (15-41)	Q4 (58-79)	Q1 (13-20)	Q4 (27-36)	Q1 (22-31)	Q4 (40-51)
n	612	194	188	118	178	147	151	147	160	145	159	154	153	159	171	176	134
Omega-3 (g/day), median (IQR)	3.56 (2.71, 4.54)	3.05 (2.42, 3.85)	4.16 (3.35, 5.06) [a]	2.92 (2.28, 3.72)	4.12 (3.35, 5.09) [a]	3.20 (2.60, 4.01)	4.07 (3.30, 5.03) [a]	3.31 (2.56, 4.09)	3.87 (2.86, 4.84) [a]	3.20 (2.46, 4.16)	3.90 (3.06, 4.92) [a]	3.04 (2.46, 4.09)	4.06 (3.14, 4.95) [a]	3.24 (2.54, 4.22)	3.71 (2.87, 4.82) [a]	3.52 (2.87, 4.46)	3.47 (2.65, 4.46)
Vitamin B6 (mg/day), median (IQR)	4.11 (3.08, 5.83)	3.91 (2.60, 6.05)	4.46 (3.60, 5.60) [a]	3.63 (1.98, 4.94)	4.63 (3.83, 6.02) [a]	3.74 (2.11, 6.52)	4.69 (3.74, 6.09) [a]	3.91 (2.20, 6.05)	4.58 (3.73, 5.78) [a]	3.98 (2.47, 5.68)	4.51 (3.73, 5.59) [a]	3.91 (2.29, 5.98)	4.54 (3.79, 5.81) [a]	3.67 (2.10, 5.98)	4.65 (3.83, 7.01) [a]	3.82 (2.35, 5.50)	4.54 (3.53, 5.97) [a]
Vitamin B12 (µg/day), median (IQR)	11.50 (8.51, 15.97)	11.01 (7.83, 15.15)	12.08 (9.00, 16.78)	10.37 (6.16, 14.69)	12.58 (9.45, 17.26) [a]	9.73 (6.04, 14.68)	12.56 (9.82, 17.28) [a]	11.22 (6.43, 15.34)	12.11 (9.18, 17.28) [a]	10.94 (7.30, 15.11)	12.43 (9.09, 17.27) [a]	10.83 (7.19, 15.21)	12.08 (9.08, 17.04)	10.82 (6.38, 15.16)	12.56 (9.45, 17.26) [a]	11.35 (7.70, 15.34)	11.65 (8.88, 16.67)
Vitamin C (mg/day), median (IQR)	173.25 (118.94, 242.95)	155.80 (99.03, 197.80)	205.53 (146.55, 276.82) [a]	130.15 (84.82, 171.77)	208.11 (150.19, 286.55) [a]	130.75 (89.72, 189.54)	212.67 (158.91, 283.10) [a]	149.51 (95.27, 191.17)	205.41 (151.71, 280.41) [a]	157.95 (100.95, 213.12)	192.85 (141.19, 277.71) [a]	151.61 (95.33, 184.80)	204.74 (147.59, 293.22) [a]	134.16 (89.72, 182.54)	211.25 (162.99, 286.55) [a]	151.31 (95.86, 214.47)	205.41 (144.43, 264.86) [a]
Vitamin D (IU/day), median (IQR)	573.38 (448.11, 884.11)	552.30 (426.85, 883.76)	606.15 (489.68, 897.50)	525.97 (329.69)	638.25 (511.23, 1030.72) [a]	500.34 (281.82, 689.00)	625.84 (500.72, 902.41) [a]	530.12 (326.48, 794.83)	617.11 (515.66, 960.79) [a]	527.96 (348.40, 669.55)	615.43 (494.51, 1096.46) [a]	526.99 (333.01, 730.53)	625.84 (497.30, 1056.37) [a]	523.65 (324.45, 669.55)	618.31 (525.45, 1021.61) [a]	567.36 (388.81, 843.12)	601.56 (482.68, 1025.41)
Folate (mg/day), median (IQR)	1034.70 (736.37, 1292.66)	977.97 (660.87, 1199.77)	1142.63 (831.28, 1418.61) [a]	840.55 (618.09, 1129.38)	1211.61 (846.19, 1413.29) [a]	836.35 (618.09, 1175.00)	1214.93 (869.95, 1395.09) [a]	905.01 (634.17, 1241.27)	1139.30 (830.35, 1404.19) [a]	911.63 (660.32, 1212.91)	1112.97 (824.27, 1348.69) [a]	944.97 (660.32, 1253.27)	1118.26 (814.67, 1350.18) [a]	846.33 (592.86, 1180.60)	1202.53 (847.22, 1419.35) [a]	836.52 (626.81, 1148.72)	1204.08 (846.36, 1444.49) [a]
Zinc (mg/day), median (IQR)	23.65 (12.64, 33.92)	22.19 (10.70, 32.67)	24.74 (14.33, 35.20)	18.40 (10.13, 32.14)	25.82 (15.14, 35.94) [a]	19.15 (10.55, 32.02)	25.82 (14.45, 36.09) [a]	19.60 (10.68, 32.56)	26.48 (14.56, 34.90) [a]	21.96 (10.91, 32.72)	24.72 (14.81, 34.30)	20.97 (10.13, 32.59)	25.39 (14.45, 34.06) [a]	16.85 (9.98, 32.21)	26.53 (15.14, 35.22) [a]	20.41 (10.68, 33.21)	24.96 (13.92, 34.23)

Data are presented as median (interquartile range). P-values were calculated using a Kruskal-Wallis test.

[a] P<0.05 for comparison of quartile 4 versus quartile 1 (reference).

Abbreviations: AHA, American Heart Association diet recommendations; AHEI, Alternate Healthy Eating Index; AMD, Alternate Mediterranean diet; DASH, Dietary Approaches to Stop Hypertension diet; IU, international units; HEI, Healthy Eating Index; IQR, interquartile range; n, sample size; PBD, Plant-based diet; PMD, Panagiotakos Mediterranean diet; Q, quartile; TMD, Trichopoulou Mediterranean diet.

© 2023 Salas-Huetos A et al. *JAMA Network Open*.

eFigure 1. Flow Chart of the Participants Analyzed in the Present Study

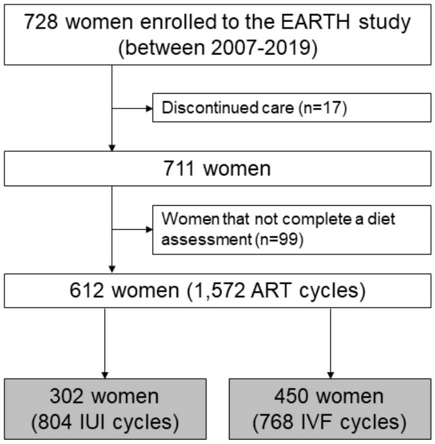

© 2023 Salas-Huetos A et al. *JAMA Network Open*.

eFigure 2. Spearman Correlations Between Adherence to the Different Dietary Patterns in (A) IVF Cycles Only, (B) IUI Cycles Only, or (C) IUI Followed by IVF Cycles

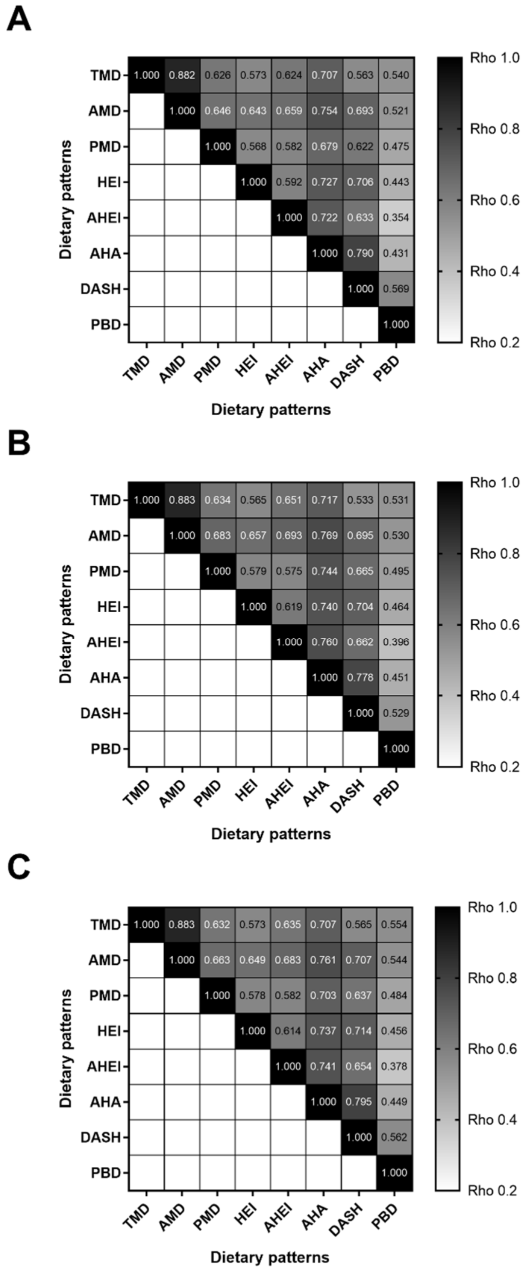

Data was presented as rho values. All correlations are statistically significant with a P-value < 0.001.

Abbreviations: AHA, American Heart Association Index; AHEI, Alternate Healthy Eating Index; AMD, Alternate Mediterranean diet; DASH, Dietary Approach to Stop Hypertension index; HEI, Healthy Eating Index; PBD, Plant-based diet; PMD, Panagiotakos Mediterranean diet; TMD, Trichopoulou Mediterranean diet.

© 2023 Salas-Huetos A et al. *JAMA Network Open*.

eReferences.

1. Trichopoulou A, Costacou T, Bamia C, Trichopoulos D. Adherence to a Mediterranean Diet and Survival in a Greek Population. *N Engl J Med*. 2003;348(26):2599-2608. doi:10.1056/NEJMoa1613303

2. Fung TT, Rexrode KM, Mantzoros CS, Manson JE, Willett WC, Hu FB. Mediterranean diet and incidence of and mortality from coronary heart disease and stroke in women. *Circulation*. 2009;119(8):1093-1100. doi:10.1161/CIRCULATIONAHA.108.816736

3. Panagiotakos DB, Pitsavos C, Arvaniti F, Stefanadis C. Adherence to the Mediterranean food pattern predicts the prevalence of hypertension, hypercholesterolemia, diabetes and obesity, among healthy adults; the accuracy of the MedDietScore. *Prev Med (Baltim)*. 2007;44(4):335-340. doi:10.1016/j.ypmed.2006.12.009

4. Krebs-Smith SM, Pannucci TRE, Subar AF, et al. Update of the Healthy Eating Index: HEI-2015. *J Acad Nutr Diet*. 2018;118(9):1591-1602. doi:10.1016/j.jand.2018.05.021

5. McCullough ML, Feskanich D, Stampfer MJ, et al. Diet quality and major chronic disease risk in men and women: Moving toward improved dietary guidance. *American Journal of Clinical Nutrition*. 2002;76(6):1261-1271. doi:10.1093/ajcn/76.6.1261

6. Rehm CD, Peñalvo JL, Afshin A, Mozaffarian D. Dietary intakes among US adults, 1999-2012 HHS Public Access. *JAMA*. 2016;315(23):2542-2553. doi:10.1001/jama.2016.7491.

7. Fung TT, Chiuve SE, McCullough ML, Rexrode KM, Logroscino G, Hu FB. Adherence to a DASH-style diet and risk of coronary heart disease and stroke in women. *Arch Intern Med*. 2008;168(7):713-720. doi:10.1001/archinte.168.7.713

8. Martínez-González MA, Sánchez-Tainta A, Corella D, et al. A provegetarian food pattern and reduction in total mortality in the Prevención con Dieta Mediterránea (PREDIMED) study. *American Journal of Clinical Nutrition*. 2014;100(SUPPL. 1):320-328. doi:10.3945/ajcn.113.071431

© 2023 Salas-Huetos A et al. *JAMA Network Open*.
