# Supplementary material for: Women’s Adherence to Healthy Dietary Patterns and Outcomes of Infertility Treatment
Source: JAMA Netw Open. 2023 Aug 18;6(8):e2329982. doi: 10.1001/jamanetworkopen.2023.29982 (PMC10439476; doi:10.1001/jamanetworkopen.2023.29982)
Supplement: Supplement 2. — Group Information. EARTH Study Team [file jamanetwopen-e2329982-s002.pdf]

Supplemental Online Content: Nonauthor Collaborators
*First name, last name, and suffix (if applicable) are required and will appear in PubMed.

*Group Name(s): EARTH Study Team

***First Name and Middle Initial(s)**	***Last Name**	***Suffix (eg, Jr, III)**	Academic Degrees	Institution	Location (city, state/province, country)	Role or Contribution, eg, chair, principal investigator	Group (if more than 1 Group listed in the byline) and/or Subgroup (eg, Steering Committee)
Russ	Hauser		MD, ScD	Harvard TH Chan School of Public Hea	Boston, MA. USA	Principal Investigator	
Paige L	Williams		PhD	Harvard TH Chan School of Public He	Boston, MA. USA	Co-Investigator; Senior Statistician	
Jorge E	Chavarro		MD, ScD	Harvard TH Chan School of Public He	Boston, MA. USA	Co-Investigator; Head Nutrition Resarch	
Lidia	Minguez-Alarcon		PhD	Brigham and Women's Hospital, and	Boston, MA. USA	Co-Investigator	
Jennifer	Ford		RN	Harvard TH Chan School of Public He	Boston, MA. USA	Research Nurse, Study Coordinator	
Myra	Keller		RN	Harvard TH Chan School of Public He	Boston, MA. USA	Research Nurse	
Ramace	Daad		BA	Harvard TH Chan School of Public He	Boston, MA. USA	Senior Research Assistant	
Irene	Souter		MD	Massachusetts General Hospital	Boston, MA. USA	Co-Investigator	
John	Petrozza		MD	Massachusetts General Hospital	Boston, MA. USA	Co-Investigator	
Thomas L	Toth		MD	Massachusetts General Hospital	Boston, MA. USA	Co-Investigator	
Diane L.	Wright		PhD	Massachusetts General Hospital	Boston, MA. USA	Co-Investigator	
Charles	Bormann		PhD	Massachusetts General Hospital	Boston, MA. USA	Co-Investigator	
