# Supplementary material for: Women’s Adherence to Healthy Dietary Patterns and Outcomes of Infertility Treatment
Source: JAMA Netw Open. 2023 Aug 18;6(8):e2329982. doi: 10.1001/jamanetworkopen.2023.29982 (PMC10439476; doi:10.1001/jamanetworkopen.2023.29982)
Supplement: Supplement 3. — Data Sharing Statement [file jamanetwopen-e2329982-s003.pdf]

Data Sharing Statement

Salas-Huetos. Women’s Adherence to Healthy Dietary Patterns and Outcomes of Infertility Treatment. *JAMA Netw Open*. Published August 18, 2023.
doi:10.1001/jamanetworkopen.2023.29982

Data
Data available: No

Additional Information
Explanation for why data not available: The datasets and SAS statistical codes generated and/or analyzed during the current study are available from the corresponding author on reasonable request.
